# Rapid drift of the Tethyan Himalaya terrane before two-stage India-Asia collision

**DOI:** 10.1093/nsr/nwaa173

**Published:** 2020-07-27

**Authors:** Jie Yuan, Zhenyu Yang, Chenglong Deng, Wout Krijgsman, Xiumian Hu, Shihu Li, Zhongshan Shen, Huafeng Qin, Wei An, Huaiyu He, Lin Ding, Zhengtang Guo, Rixiang Zhu

**Affiliations:** State Key Laboratory of Lithospheric Evolution, Institute of Geology and Geophysics, Chinese Academy of Sciences, Beijing 100029, China; Innovation Academy for Earth Science, Chinese Academy of Sciences, Beijing 100029, China; College of Earth and Planetary Sciences, University of Chinese Academy of Sciences, Beijing 100049, China; College of Resources, Environment and Tourism, Capital Normal University, Beijing 100048, China; State Key Laboratory of Lithospheric Evolution, Institute of Geology and Geophysics, Chinese Academy of Sciences, Beijing 100029, China; Innovation Academy for Earth Science, Chinese Academy of Sciences, Beijing 100029, China; College of Earth and Planetary Sciences, University of Chinese Academy of Sciences, Beijing 100049, China; Department of Earth Sciences, Utrecht University, Utrecht HD 3584, The Netherlands; State Key Laboratory of Mineral Deposits Research, School of Earth Sciences and Engineering, Nanjing University, Nanjing 210029, China; State Key Laboratory of Lithospheric Evolution, Institute of Geology and Geophysics, Chinese Academy of Sciences, Beijing 100029, China; State Key Laboratory of Lithospheric Evolution, Institute of Geology and Geophysics, Chinese Academy of Sciences, Beijing 100029, China; Innovation Academy for Earth Science, Chinese Academy of Sciences, Beijing 100029, China; College of Earth and Planetary Sciences, University of Chinese Academy of Sciences, Beijing 100049, China; State Key Laboratory of Lithospheric Evolution, Institute of Geology and Geophysics, Chinese Academy of Sciences, Beijing 100029, China; Innovation Academy for Earth Science, Chinese Academy of Sciences, Beijing 100029, China; State Key Laboratory of Mineral Deposits Research, School of Earth Sciences and Engineering, Nanjing University, Nanjing 210029, China; State Key Laboratory of Lithospheric Evolution, Institute of Geology and Geophysics, Chinese Academy of Sciences, Beijing 100029, China; Innovation Academy for Earth Science, Chinese Academy of Sciences, Beijing 100029, China; College of Earth and Planetary Sciences, University of Chinese Academy of Sciences, Beijing 100049, China; College of Earth and Planetary Sciences, University of Chinese Academy of Sciences, Beijing 100049, China; Key Laboratory of Continental Collision and Plateau Uplift, Institute of Tibetan Plateau Research, Chinese Academy of Sciences, Beijing 100101, China; Innovation Academy for Earth Science, Chinese Academy of Sciences, Beijing 100029, China; Key Laboratory of Cenozoic Geology and Environment, Institute of Geology and Geophysics, Chinese Academy of Sciences, Beijing 100029, China; CAS Center for Excellence in Life and Paleoenvironment, Beijing 100029, China; State Key Laboratory of Lithospheric Evolution, Institute of Geology and Geophysics, Chinese Academy of Sciences, Beijing 100029, China; Innovation Academy for Earth Science, Chinese Academy of Sciences, Beijing 100029, China; College of Earth and Planetary Sciences, University of Chinese Academy of Sciences, Beijing 100049, China

**Keywords:** India-Asia collision, Tethyan Himalaya terrane, North India Sea, two-stage continental collision

## Abstract

The India-Asia collision is an outstanding smoking gun in the study of continental collision dynamics. How and when the continental collision occurred remains a long-standing controversy. Here we present two new paleomagnetic data sets from rocks deposited on the distal part of the Indian passive margin, which indicate that the Tethyan Himalaya terrane was situated at a paleolatitude of ∼19.4°S at ∼75 Ma and moved rapidly northward to reach a paleolatitude of ∼13.7°N at ∼61 Ma. This implies that the Tethyan Himalaya terrane rifted from India after ∼75 Ma, generating the North India Sea. We document a new two-stage continental collision, first at ∼61 Ma between the Lhasa and Tethyan Himalaya terranes, and subsequently at ∼53−48 Ma between the Tethyan Himalaya terrane and India, diachronously closing the North India Sea from west to east. Our scenario matches the history of India-Asia convergence rates and reconciles multiple lines of geologic evidence for the collision.

## INTRODUCTION

**Figure 1.**
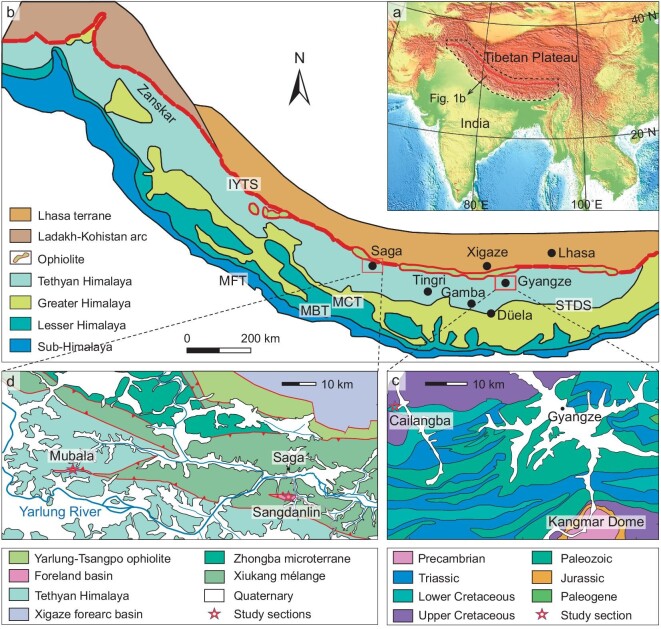
Geologic and topographic maps of the study region. (a) Large-scale topographic map. (b) The geology of the Himalaya, simplified from Yin [7]. (c) The studied Cailangba section, simplified from Chen *et al.* [32]. (d) The studied Sangdanlin and Mubala sections. IYTS, Indus-Yarlung Tsangpo Suture. STDS, South Tibet Detachment System. MCT, Main Central Thrust. MBT, Main Boundary Thrust. MFT, Main Frontal Thrust.

The collision of India with Asia was one of the most significant tectonic events of Earth's history and had a profound influence on deep physical and chemical processes, paleogeography, climate and biodiversity in Asia [1–6] (Fig. 1). During the past half century, a series of geologic, geophysical and geochemical studies [7–14] has contributed significantly to our understanding of the timing and process of the India-Asia collision. As a result, chronological consensus exists that Asia-derived sediments were deposited in the Tethyan Himalaya at ∼60 Ma, during the mid-Paleocene [15–17], which is generally accepted as the initiation time of India-Asia collision [9–14]. At least four competing geodynamic models were proposed to discuss the dynamic process of the India-Asia collision (Supplementary Fig. 1): (i) the continental Greater India model [18,19] successfully explains the arrival of Asian sediments in the Tethyan Himalaya at ∼60 Ma, but the required ∼4500 km of post-collisional convergence is significantly more than the shortening in the geologic records; (ii) the Greater India Basin model [5,12] fulfils paleomagnetic and plate kinematic criteria, explains the shortening, accommodates ∼60 Ma Tethyan Himalaya-Lhasa collision, but invokes the opening and closure of an oceanic basin between ∼120 Ma and ∼25 Ma between the Tethyan Himalaya and India, of which there is no accretionary geologic record present in the Himalaya; (iii) the island arc-continent collision model [20] invokes an always small Greater India which satisfies existing paleomagnetic, plate kinematic criteria and fast India-Asia convergence but fails to explain why Asian detritus reaches the Tethyan Himalaya at ∼60 Ma; and (iv) the India-arc collision with the Xigaze backarc basin model [13] has an always narrow Greater India constrained by existing paleomagnetic and plate kinematic data, which successfully explains the arrival of Asian sediments in the Tethyan Himalaya at ∼60 Ma, but invokes the opening and closure of a backarc basin between the Tethyan Himalaya and Lhasa terrane of which there is no geologic record. In spite of the competing character of these models, precise determination of the timing and process of the continental collision between India and Asia is required to provide reliable and independent evidence.

Paleomagnetism can effectively quantify paleolatitudes of plates [21] and has been widely used to quantify the India-Asia collision process. Paleomagnetic studies indicate that the Lhasa terrane has been relatively stable in its location since the Early Cretaceous at paleolatitudes of 10°N–20°N [22–24]. In contrast, the paleomagnetic data from Upper Cretaceous to Paleocene rocks of the Tethyan Himalaya terrane suggest variable paleolatitudes (15°S–10°N) [25–28]. Conflicting results are partly due to the remagnetization of limestones in some previous studies, as suggested by Huang *et al*. [29].

To this end, we conducted paleomagnetic and rock magnetic analyses on two key successions that were deposited on the distal northern part of the Indian passive margin (Tethyan Himalaya terrane), where Upper Cretaceous oceanic red beds (CORBs) are exposed in the Cailangba A and B sections (28.9°N, 89.2°E) in the Gyangze area and Upper Cretaceous to Paleocene red siliceous shales are well-exposed and well-studied in the Sangdanlin (29.3°N, 85.3°E) and Mubala (29.3°N, 84.7°E) sections in the Saga area [15–17,30–32] (Fig. 1, Supplementary Note 1 and Supplementary Figs 2 and 3). All these strata were interpreted to be deposited on the lower continental slope, representing the most distal northern continental margin of India. Our results provide independent evidence of the paleolatitudinal positions of the Tethyan Himalaya terrane in the late Cretaceous and mid-Paleocene and unequivocally elucidate the timing, location and geodynamic models of the India-Asia collision.

## RESULTS

### Scanning electron microscopy

**Figure 2.**
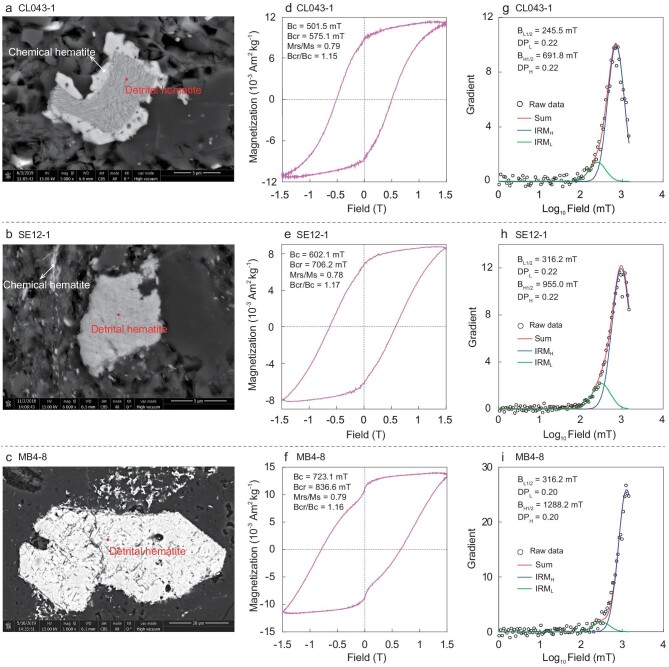
SEM observations and rock magnetic properties from the Cailangba (sample CL043-1), Sangdanlin (sample SE12-1) and Mubala (sample MB4-8) sections. (a−c) Scanning electron microscopy backscatter electron images. (d−f) Hysteresis loops after high-field slope correction with hysteresis parameters indicated. (g−i) Component analysis of coercivity distributions with green, blue and red lines indicating the low coercivity (IRM_L_) component, high coercivity (IRM_H_) component and the sum of these components (sum), respectively. Open circles indicate raw IRM gradient data (raw data).

Two different types of hematite grains were found in the thin sections of the Cailangba and Sangdanlin samples (Fig. 2a and b). One type has a particle size range of 5−15 μm with rectangular, triangular, subcircular and irregular shapes. This type of hematite is interpreted as detrital in origin. The other type has a particle size range of ∼1 μm, presenting a subhedral to euhedral regular morphology, either distributed along fractures or grown in pores. This type of hematite is interpreted as chemical (authigenic) in origin. We conclude that the magnetic mineral assemblage of these samples consists of both detrital and chemical hematite grains. The Mubala samples reveal only the large particle component and contain only detrital hematite grains (Fig. 2c).

### Rock magnetism

All the selected samples display rectangular hysteresis loops (Fig. 2d−f), which are typical of hematite. The isothermal remanent magnetization (IRM) acquisition curves show that the magnetic remanence is not saturated at the maximum applied field of 1.5 T. The remanent coercivity (Bcr) values (defined with respect to 1.5 T) are as large as 575−837 mT. All these results are characteristic of a magnetization dominated by high-coercivity hematite. However, component analyses of coercivity distributions [33] show different assemblages of hematite grains.

For the Cailangba and Sangdanlin samples, two components with different coercivities were distinguished by the IRM component analysis (Fig. 2g and h). Component 1 with median acquisition field (B_L1/2_) of 246−316 mT, which contributes about 15% to the saturation isothermal remanent magnetization (SIRM), is interpreted as fine-grained chemical hematite. Component 2 with median acquisition field (B_H1/2_) of 692−955 mT, which contributes about 85% to SIRM, is interpreted as coarse-grained detrital hematite. For the Mubala samples, the predominant component with median acquisition field (B_H1/2_) of up to 1288 mT (Fig. 2i), which contributes 91% to the SIRM, is also interpreted as coarse-grained detrital hematite.

### Paleomagnetism

**Figure 3.**
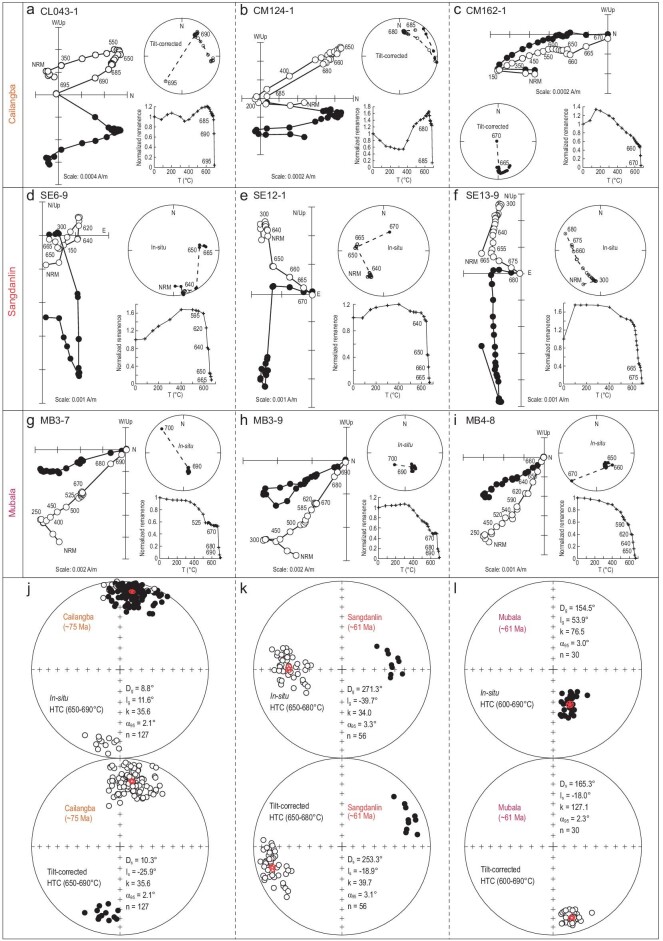
Paleomagnetic results. (a−i) Representative orthogonal demagnetization diagrams in geographic (stratigraphic) coordinates with corresponding normalized natural remanent magnetization (NRM) vs. temperature plots and equal-area stereonets in the Cailangba (specimens CL043-1, CM124-1 and CM162-1), Sangdanlin (specimens SE6-9, SE12-1 and SE13-9) and Mubala (specimens MB3-7, MB3-9 and MB4-8) sections. (j−l) Equal-area projections of *in situ* (upper) and tilt-corrected (lower) paleomagnetic directions. (j) HTC directions of all specimens in the Cailangba section. (k) HTC directions of all specimens in the Sangdanlin section. (l) HTC directions of all specimens in the Mubala section. Red circles around the red stars in (j−l) denote the 95% confidence limit and mean directions. Solid and open symbols denote the lower and upper hemisphere projections, respectively.

Stepwise thermal demagnetization reveals three magnetic components for specimens of the Cailangba and Sangdanlin sections (Fig. 3a–f): a low-temperature component (LTC, 80–300°C), a middle-temperature component (MTC, 300–650°C) and a high-temperature component (HTC, 650–680°C); and only two components for specimens of the Mubala section (Fig. 3g–i): an LTC (80–300°C) and an HTC (600–680°C).

**Figure 4.**
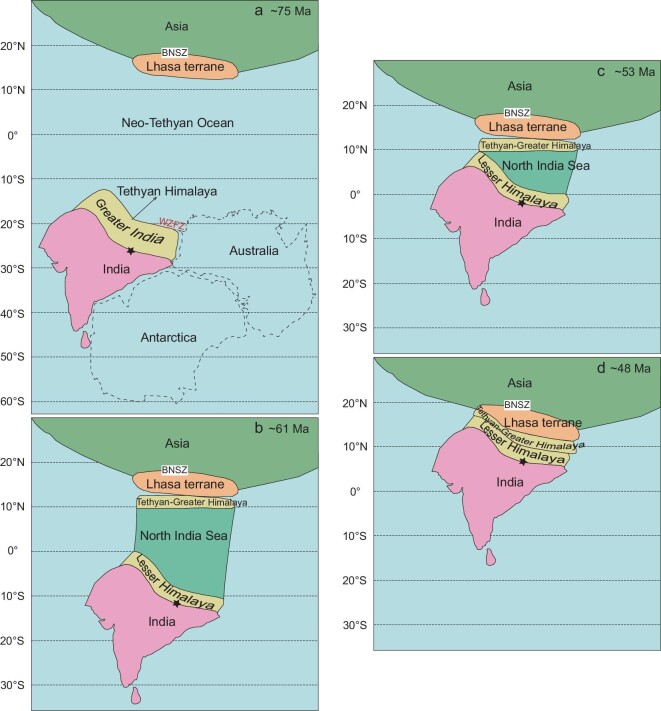
Geodynamic evolution of the India-Asia continental collision showing the paleogeographic patterns: (a) ∼75 Ma, (b) ∼61 Ma, (c) ∼53 Ma and (d) ∼48 Ma. Our ∼75-Ma paleogeographic pattern (a) is consistent with the paleogeographic reconstructions of the western margin of the Australian continent based on the sub-continent's pre-breakup position within Gondwana and on the present-day bathymetry of the Indian Ocean to the west of Australia [67]. The Australian and Antarctic continents in (a) were reconstructed in the Gondwana framework only for determining the size of eastern Greater India. BNSZ, Bangong-Nujiang suture zone. WZFZ, Wallaby-Zenith fracture zone. The black stars indicate the reference point (29.3°N, 85.3°E) when calculating the extension of Greater India in this study.

The sample-mean directions of the LTCs before tilt correction are close to the present geomagnetic field direction of the sampling area, indicating that the LTCs are of viscous origin (Table 1 and Supplementary Figs 4*−*6). The MTCs were isolated from the Cailangba and Sangdanlin sections (Table 1 and Supplementary Figs 4 and 5). The HTCs were isolated from 127, 56 and 30 specimens at the Cailangba, Sangdanlin and Mubala sections, respectively (Fig. 3j−l, Table 1 and Supplementary Table 1).

**Table 1.** Mean directions. N, number of samples used to calculate mean direction; D_g_ and I_g_ (D_s_ and I_s_), directions in geographic (stratigraphic) coordinates; k_g_/k_s_, best estimate [66] precision parameter for the mean direction in geographic (stratigraphic) coordinates; α_95g_/α_95s_, the radius of cone at 95% confidence level about the mean direction in geographic (stratigraphic) coordinates.

Sections	Magnetic components	N	D_g_	I_g_	k_g_	α_95g_	D_s_	I_s_	k_s_	α_95s_
Cailangba	LTC, 80–300°C	185	1.6	42.4	40.3	1.7	358.5	4.7	41.6	1.6
	MTC, 300–650°C	165	162.3	−18.5	28.5	2.1	162.0	18.4	21.6	2.4
	HTC, 650–680°C	127	8.8	11.6	35.6	2.1	10.3	−25.9	35.6	2.1
Sangdanlin	LTC, 80–300°C	101	358.6	40.8	19.4	3.3	5.3	7.0	20.7	3.2
	MTC, 300–650°C	96	180.6	−16.1	70.0	1.7	180.6	16.9	69.1	1.7
	HTC, 650–680°C	56	271.3	−39.7	34.0	3.3	253.3	−18.9	39.7	3.1
Mubala	LTC, 80–300°C	31	3.1	46.4	19.2	6.1	179.2	57.5	17.6	6.3
	HTC, 600–680°C	30	154.5	53.9	76.5	3.0	165.3	−18.0	127.1	2.3

The HTCs were not isolated from some individual specimens of the Sangdanlin section (Supplementary Fig. 7). In these cases, it is possible that a chemical remanent magnetization, generated through oxidation of magnetite by orogenic hydrothermal fluids, has overprinted the primary detrital remanent magnetization completely [34,35]. The HTCs are considered the primary magnetization in all our sections; they are carried by detrital hematite and show dual polarities (Supplementary Figs 2 and 3).

### Reliability of the paleomagnetic data

For the Cailangba A and B sections, the fold tests failed because the strata at two sections are uniformly dipping. However, the HTCs from the Cailangba B section display both normal and reverse polarities (Supplementary Fig. 2 and Supplementary Table 1), which pass the bootstrap reversal test (B class) [36]. The HTC sample-mean declinations of the Sangdanlin section differ significantly from those of the Mubala section, but the inclinations trend to the same value after tilt correction (Fig. 3 and Supplementary Fig. 8). Using the method of maximum likelihood estimation [37] to calculate the sample-mean inclination, the precision parameter k value increased from 26.9 (*in situ*) to 82.8 after tilt correction, indicating that the remanences have a pre-folding origin. Although separated by 60 km, the Sangdanlin Formation strata at the two studied sections reveal the same sedimentary facies. The anisotropy of magnetic susceptibility result suggests that the Sangdanlin section experienced 71° counterclockwise rotation with respect to the Mubala section (Supplementary Note 2 and Supplementary Fig. 9b and d). After correction for this local rotation, positive fold tests are achieved for the sample-mean direction of these two sections, yielding D_s_ = 176.3°, I_s_ = −18.8°, k = 35.8, α_95_ = 2.6°, N = 86 after tilt correction (Supplementary Note 3 and Supplementary Fig. 10). Moreover, the dual polarity data of the Sangdanlin section (Supplementary Fig. 3 and Supplementary Table 1) also pass the reversal test (B class) [36]. All these lines of evidence suggest that the mean directions of HTCs of the Cailangba, Sangdanlin and Mubala sections are of pre-folding origin and most likely primary.

### Magnetostratigraphy

The HTCs in the Cailangba and Sangdanlin sections display both normal and reverse polarities, which we use to construct magnetic polarity sequences of the sections that can be subsequently correlated to the geomagnetic polarity time scale (GPTS) [38] to better estimate the age of the rocks (Supplementary Figs 2 and 3 and Supplementary Table 1). The magnetic polarity zones correlate very well with the GPTS, which also implies a primary origin of the magnetic remanences.

The lower and middle parts of the Cailangba B section were dated to be 76.2–75.7 Ma by planktonic foraminifers, which yielded a middle Campanian age for the Cailangba B section (Supplementary Fig. 2) [32]. Hence, the reverse polarity R1 and normal polarity N2 magnetozones in the Cailangba B section are correlated to chron C32r.2r (74.3–74.0 Ma) and the upper part of chron C33n (79.9–74.3 Ma), respectively. The single normal polarity N1 magnetozone of the Cailangba A section is correlated to the upper part of chron C33n (79.9–74.3 Ma). As a result, the CORBs in the Cailangba A and B sections represent the time interval of 76.2–74.0 Ma by magnetobiostratigraphy (Supplementary Fig. 2 and Supplementary Table 1).

For the Sangdanlin section, biostratigraphic constraints are available from sub-units 9−13 that were dated to be in Paleogene radiolarian zones RP4–RP6 (62.8−56.9 Ma) at low latitude (Supplementary Fig. 3) [17]. The nannofossil biostratigraphy of the overlying Zheya Formation corresponds to the upper part of the Paleocene calcareous nannofossil zone 7 (CNP7: 59.93−58.27 Ma), and correlates robustly with the upper part of chron C26r of Ocean Drilling Program Site 1262 [17,39]. Detrital zircon geochronology data from sub-units 14–16 show the youngest peak at 58.1 ± 0.9 Ma (Supplementary Fig. 3) [17], which is consistent with the detrital zircon age results (∼60−59 Ma) reported by DeCelles *et al*. [15] and Wu *et al*. [16]. Based on these independent age constraints, we correlate normal polarity N1 magnetozone to chron C27n (62.5−62.2 Ma) and reverse polarity R1 magnetozone to chron C26r (62.2−59.2 Ma), respectively (Supplementary Fig. 3 and Supplementary Table 1). As a result, the Sangdanlin Formation is interpreted to have been deposited during 62.5−59.2 Ma.

### Inclination shallowing test and correction

Inclination shallowing may be induced by depositional and post-depositional compaction in red beds and other sediments [40]. The sampled beds of late Cretaceous Cailangba A and B sections are uniform and the amount of sampled beds exceeds 100. Elongation/inclination (E/I) correction [40] and IRM anisotropy-based inclination shallowing correction [41] are independent, but both yield a shallowing factor of ∼0.7 (Supplementary Note 4, Supplementary Figs 11 and 12, and Supplementary Table 2). After inclination shallowing correction, the sample-mean inclination of the Cailangba section increased from 25.9° to 35.0°, giving what we propose to be a high-quality Late Cretaceous paleopole of 40.8°N/256.3°E, A_95_ = 1.8°, with a paleolatitude of 19.4° ± 1.8°S.

Considering the number of sampled beds of the Sangdanlin section, only IRM anisotropy-based inclination shallowing correction [41] was used for the red siliceous shales of the Sangdanlin and Mubala sections. Using the anisotropy-based mean value of ∼0.7 as the inclination shallowing factor (Supplementary Note 4, Supplementary Fig. 13 and Supplementary Table 3), the sample-mean inclination of the Sangdanlin and Mubala sections (Supplementary Fig. 10) increased from 18.8° to 26.0°, giving a paleopole of 74.0°N/278.5°E, A_95 _= 2.5°, with a paleolatitude of 13.7° ± 2.5°N.

## DISCUSSION

### Primary versus secondary magnetizations in the Tethyan Himalaya terrane

Attempts at paleomagnetic reconstructions for the India-Asia collision zone are commonly hampered by rocks that contain secondary remagnetization overprints, but that these data have been interpreted to be primary [29]. We show that red rocks of the Tethyan Himalaya terrane contain both secondary components, carried by chemical hematite (our MTC component), and primary components, carried by detrital hematite (our HTC component).

Recently Yang *et al*. [42] also reported paleomagnetic results from red beds in the Sangdanlin section, with a direction (defined through 500−660°C) of D_g_ = 177.0°, I_g_ = −14.1°, k = 19.4, α_95_ = 5.6° before tilt correction, and D_s_ = 178.7°, I_s_ = +9.5°, k = 20.8, α_95_ = 5.4° after tilt correction. This is similar to the sample-mean direction of the MTCs carried by secondary chemical hematite (defined through 300−650°C) of the Sangdanlin specimens in this study (Table 1 and Supplementary Fig. 5), and thus, we argue, cannot be used to reconstruct the India-Asia collision. The MTCs were probably acquired due to the folding and thrusting during a later tectonic episode of the Himalayan orogeny.

### Timing and position of the collision between the Tethyan Himalaya and Lhasa terranes

Although numerous paleomagnetic studies have been conducted on both the Lhasa and Tethyan Himalaya terranes over the past decades, the derived paleolatitudes remain highly variable and thus controversial. In this study, we evaluate the Cretaceous and Paleogene paleomagnetic data from the Lhasa terrane based on the stringent criteria proposed by van der Voo [43]. We especially focus on data from red beds with inclination shallowing properly corrected, and from volcanic rocks with secular variation (PSV) averaged. A total of 19 paleomagnetic poles (five for Early Cretaceous, eight for Late Cretaceous and six for Paleogene time) passed these criteria and were selected to estimate paleolatitude changes of the Lhasa terrane from Cretaceous to Paleogene (Supplementary Fig. 14 and Supplementary Table 4). The small-circle fitting method [44] was applied to fit the selected Lhasa terrane paleomagnetic poles at a reference point (29.3°N, 85.3°E).

Our review of the Lhasa data accepts five reliable Early Cretaceous poles and eight Late Cretaceous poles that give co-latitudes of 75.9° ± 5.9°N and 76.6° ± 2.5°N, respectively, indicating paleolatitudes of 14.1° ± 5.9°N in the Early Cretaceous and 13.4° ± 2.5°N in the Late Cretaceous for the Lhasa terrane; these are equivalent within errors. In contrast, paleomagnetic directions of some Upper Cretaceous and Paleocene limestones (Zongpu and Zongshan formations) yield lower paleolatitudes, ranging from 15°S to 10°N [25–28] (Supplementary Table 4). We hypothesize that these anomalous data are probably the result of a secondary chemical remanent magnetization [29]. In conclusion, the combined 13 reliable Cretaceous poles give a co-latitude of 76.3° ± 2.2°N, indicating a paleolatitude of 13.7° ± 2.2°N in the Cretaceous for the Lhasa terrane (Supplementary Fig. 14a).

**Figure 5.**
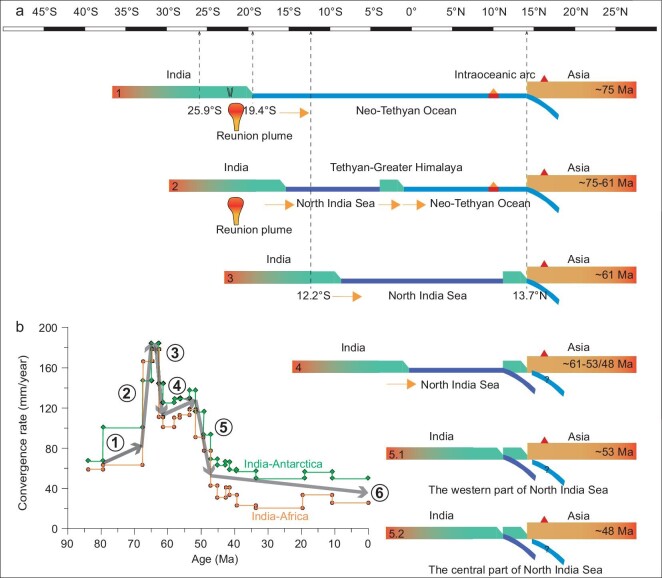
Paleomagnetically quantified India-Asia convergence history. (a) Schematic diagram showing the evolution of the proposed North India Sea and of the India-Asia continent collision. (b) India-Asia convergence rates documented by the relative plate motions between India-Africa and India-Antarctica [47], which were evaluated as the most accurate recordings of India's plate motion [52]. The numbers with arrows in (b) roughly correspond to the stages in (a). Our new paleomagnetic results from the CORBs in the Tethyan Himalaya yield a paleolatitude of 19.4° ± 1.8°S at ∼75 Ma, while a paleolatitude of 25.9° ± 2.9°S is obtained by the APWP of India at ∼75 Ma (also see Fig. 4a). Our new paleomagnetic results indicate that the Tethyan Himalaya terrane was situated at a paleolatitude of 13.7° ± 2.5°N at ∼61 Ma, while a paleolatitude of 12.2° ± 2.1°S was inferred from APWP of India at ∼61 Ma for the same reference point (29.3°N, 85.3°E) (also see Fig. 4b).

Our new paleomagnetic results from the Cailangba section indicate that the Tethyan Himalaya terrane was at a paleolatitude of 19.4° ± 1.8°S during the Late Cretaceous at a reference point (29.3°N, 85.3°E) (Figs 4 and 5). This indicates that it was separated from the Lhasa terrane by 33.1° ± 2.8°, equivalent to some 3641 ± 308 km.

Moreover, our literature review provides six reliable Paleogene poles resulting in a co-latitude of 74.3° ± 5.3°N, indicating a paleolatitude of 15.7° ± 5.3°N for the Lhasa terrane in the Paleogene (Supplementary Fig. 14b). This is similar to the Cretaceous result for the Lhasa terrane and we conclude that the southern margin of the Asian plate remained in a relatively stable position since the Early Cretaceous.

Our new paleomagnetic results provide so far the most reliable and accurate constraint on the paleolatitude of the northern margin of the Indian continent, indicating a position at 13.7° ± 2.5°N during the mid-Paleocene. This demonstrates that the paleolatitude of the Tethyan Himalaya terrane overlapped within errors with that of the Lhasa terrane, supporting the hypothesis that the continental collision between the Tethyan Himalaya and Lhasa terranes must have occurred at a paleolatitude of ∼14°N at ∼61 Ma (Figs 4 and 5).

### Geodynamics of the India-Asia collision

The paleolatitudinal difference of the Tethyan Himalaya terrane in the Late Cretaceous (19.4° ± 1.8°S) and the mid-Paleocene (13.7° ± 2.5°N) indicates an anomalously high speed of ∼260.1 mm/year during the interval ∼75 Ma to ∼61 Ma. A much slower speed of ∼99.6 mm/year was obtained for India by calculating the apparent polar wander path (APWP) of India during the interval 80−60 Ma for the same reference point (29.3°N, 85.3°E) [45]. This large magnitude difference of northward motion implies that after ∼75 Ma the Tethyan Himalaya terrane rifted away from India. The rifting may have been induced by the melting of the lower half of the Indian passive continental margin lithosphere with upwelling of the Reunion plume [46,47]. This plume is currently located at the latitude of ∼21°S, very close to the paleolatitude of 19.4° ± 1.8°S for the distal Indian passive continental margin at ∼75 Ma. In addition, this fast terrane movement may have been further accelerated by the long-time subducting slab of Neo-Tethyan oceanic lithosphere beneath the southern part of the Asian continent, which resulted in metamorphism of basaltic rock to eclogite facies in the lower lithosphere [48,49] and hence dragged the Tethyan Himalaya from India. The combined effects of the above-mentioned processes likely generated a pull-apart basin during ∼75−61 Ma, which we invoke as the ‘North India Sea’ (Figs 4 and 5). This scenario is slightly different from the Greater India Basin model, in which a basin was formed by N-S extension in the early Cretaceous [5,12].

Our new paleomagnetic data indicate that the first collision between Tethyan Himalaya and Lhasa occurred at ∼61 Ma at a paleolatitude of ∼14°N. Considering that the paleolatitude of the southern Asian margin remained almost constant from early Cretaceous to the Paleogene (Supplementary Fig. 14), the central part of the North India Sea had a latitudinal width of 2134 ± 521 km (19.4° ± 4.7°) at ∼61 Ma (Fig. 4b), which is estimated by the size difference of Greater India between ∼75 and ∼61 Ma for the reference point (29.3°N, 85.3°E). In terms of tectonic structure, there is a paleolatitudinal difference of 7.6° between the western and central parts of the Indian passive margin, which is estimated based on the paleolatitude difference obtained by the APWP of India for the reference points (28.3°N, 85.3°E) and (34.5°N, 73.3°E) at ∼61 Ma [45]. The western part of the North India Sea had a latitudinal width of 1298 ± 521 km (11.8° ± 4.7°) at ∼61 Ma (Fig. 4b). Then India continued to drift northward. Based on India's northward drift rates, calculated from its APWP during the intervals 70−60 Ma, 60−50 Ma and 50−40 Ma [45], it took 7.6 + 3.0/−2.9 Myr to close the western part and 13.3 + 4.7/−3.8 Myr to close the central part of the North India Sea. The second collision between India and Tethyan Himalaya from its western part to central part is thus expected to have occurred diachronously between ∼53 Ma and ∼48 Ma (Figs 4c and d).

Our proposed North India Sea hypothesis and associated two-stage continental collision between India and Asia consistently explain the history of the India-Asia convergence rates (Fig. 5), which were established by the relative plate motions between India-Africa and India-Antarctica [47] that show five phases of the convergence history. For example, the rapid acceleration from ∼80 mm/year at ∼70 Ma to ∼180 mm/year at ∼63 Ma (phase 2) is roughly synchronous with the opening of the North India Sea due to upwelling of the Reunion plume [46,47], and the subsequent rapid slowdown from ∼180 mm/year at ∼63 Ma to ∼110 mm/year at ∼61 Ma (phase 3) is roughly synchronous with the progressive closure of the Neo-Tethyan Ocean and the convergence between Tethyan Himalaya and Asia. The moderate acceleration from ∼110 mm/year at ∼61 Ma to ∼130 mm/year at ∼53 Ma (phase 4) corresponds to the shrinkage of the North India Sea, and the noticeable slowdown from ∼130 mm/year at ∼53 Ma to ∼50 mm/year at ∼48 Ma (phase 5) is fully concordant with the diachronous closure of the North India Sea from its western to central parts (Fig. 4c and d and Fig. 5).

Our scenario generally agrees with the two-stage closure of a double subduction system, that is, an island arc-continent collision followed by the subsequent arc/continent complex-continent collision [50−52] consistent with the history of India-Asia convergence rates. However, our paleomagnetic data suggest that the island arc-continent collision should have occurred before ∼61 Ma, which is supported by the suggestions that the Kohistan arc had attached to Asia before or at ∼70 Ma [12,13].

Multiple lines of geologic evidence from the Tibetan Plateau support the ∼53−48 Ma-collision between the Tethyan Himalaya and India. For example, the northernmost sub-Himalaya terrane acquired detritus derived from Asia at ∼55 Ma, suggested by detrital zircon U-Pb geochronology from the lower Eocene Margalla Hill Limestone of northern Pakistan [53], and the central part of Lesser Himalaya terrane acquired detritus derived from the Tethyan Himalayan thrust belt at no later than ∼45 Ma [54,55]. Results of both studies are in good agreement with the diachronous collision scenario. Our collision model also explains the gradual cessation of marine sedimentation from west to east in the Tethyan Himalaya (52–50 Ma in the Zanskar area; 43–41 Ma in the Tingri-Gamba area and ∼35 Ma in the Düela area) (Fig. 1b) [10,56].

Independent evidence from Eocene−Oligocene rocks of the Tethyan Himalaya showing crustal shortening, rapid exhumation and magmatism, and the Greater Himalaya documenting deep tectonic burial, high-grade metamorphism and anatexis, and the Indian craton experiencing earliest foreland basin development, connects the Tethyan Himalaya with the northern Indian craton at ∼48 Ma [11,15]. Moreover, the ∼53−48 Ma-collision age would also explain the ∼30° clockwise rotation between 52 and 48 Ma in the Gonjo Basin, east-central Tibet [57], and the significant enhancement of sediment accumulation rates at ∼52−48 Ma in the Gonjo Basin [57] and at ∼54−52 Ma in the Hoh Xil Basin, north-central Tibet [58]. The ∼53−48 Ma collision and subsequent continuous convergence between India and Asia may also have caused Himalayan uplift and associated erosional exhumation, explaining the lack of Eocene- to Oligocene-age sedimentary rocks in the Himalayan region [7,10]. Finally, the ∼53−48 Ma-collision clarifies the major switch in sedimentation pattern over the Bengal Basin, characterized by a rapid increase in the influx of detritus from the Himalayas in the middle Eocene [59].

Shallow-marine strata of late Cretaceous−Eocene or Paleocene−Eocene ages, widely distributed along the Lesser Himalaya in Pakistan [53,60,61], India [62,63], Nepal [54,55] and the eastern Himalaya [64], provide direct geologic evidence for the existence of the North India Sea. The Main Central Thrust zone could be the most logical preserved location for the ancient rifting zone and the required oceanic subduction zone. Nevertheless, more multidisciplinary evidence from geologic, geophysical and geochemical investigations is required to strengthen the abovementioned geodynamic hypothesis and to accurately and precisely delineate the spatio-temporal pattern of the India-Asia collision process.

## CONCLUSION

Our new paleomagnetic data have placed the Tethyan Himalaya terrane, the northern margin of the Indian plate, at paleolatitudes of ∼19.4°S and ∼13.7°N during the intervals 76.2−74.0 Ma and 62.5−59.2 Ma, respectively. This implies the Tethyan Himalaya moved northward with an anomalously high speed of ∼260.1 mm/year, much faster than the Indian craton, which experienced a speed of ∼99.6 mm/year during the same time period. We hypothesize that the Tethyan Himalaya terrane rifted from India after ∼75 Ma, generating the North India Sea. The northward drifting Tethyan Himalaya terrane collided with Asia at ∼61 Ma, and then amalgamated with India with a diachronously closing North India Sea between ∼53 Ma and ∼48 Ma. This new two-stage collision hypothesis between India and Asia provides crucial constraints for continental collision dynamics, the uplift and deformation history of the Tibetan Plateau, and paleogeography and biodiversity patterns in Asia. Furthermore, our new findings provide key boundary conditions for climate models linking Himalaya-Tibetan orogenesis with global climate change.

## METHODS

### Sampling

We collected samples from the Cailangba A and B sections in the Gyangze area, and the Sangdanlin and Mubala sections in the Saga area using a gasoline-powered portable drill. A total of 230 samples were drilled from the Chuangde Formation CORBs in the Cailangba A and B sections with sampling intervals of 0.1−0.3 m; 130 samples from 13 sites, from fine-grained red siliceous shales and cherts in sub-units 12–13 of the Sangdanlin Formation in the Sangdanlin section; and 43 samples from four sites, from fine-grained siliceous shales and cherts of the Sangdanlin Formation in the Mubala section. To test the possibility of depositional and/or compaction-induced inclination shallowing in the red beds, five and four hand samples oriented on the strata bedding were collected from the Cailangba and Sangdanlin sections, respectively. All core samples were oriented with magnetic compass and sun compass, and then were cut into standard specimens with a length of 2.2 cm in the laboratory for stepwise thermal demagnetization. The remaining samples were used for rock magnetic measurements and scanning electron microscopy (SEM) observations.

### SEM observation

To better constrain the origin and microstructure of the magnetic minerals, polished thin sections were observed using optical microscopy and SEM. Backscattered electron microscopy analyses were conducted using a scanning electron microscope (FEI Nova NanoSEM 450) with an energy dispersive spectrometer (Oxford X-MAX80).

### Rock magnetism

In order to determine the magnetic remanence carriers in the CORBs of the Cailangba section, and the red siliceous shales of the Sangdanlin and Mubala sections, representative samples were selected for rock magnetic measurements, including hysteresis loops, IRM acquisition curves and backfield demagnetization curves with a Princeton Measurements Corporation MicroMag 3900 vibrating sample magnetometer up to a maximum field of 1.5 T. Magnetic components were analyzed using the methods of unmixing [33].

### Demagnetization of the natural remanent magnetization

All specimens were subjected to stepwise thermal demagnetization up to 670−690°C at 25−50°C intervals from room temperature to 600°C and at 5−10°C intervals from 600°C to 670−690°C using a PGL-100 thermal demagnetizer developed at the Paleomagnetism and Geochronology Laboratory (PGL). The remanence measurements were made using a 2-G Enterprises Model 755 cryogenic magnetometer installed in a magnetically shielded space with background field of <300 nT. Demagnetization results were evaluated by principal component analysis [65]. The mean directions were computed using classic Fisher statistics [66].

## Supplementary Material

nwaa173_Supplemental_File
