# Supplementary material for: Rapid drift of the Tethyan Himalaya terrane before two-stage India-Asia collision
Source: Natl Sci Rev. 2020 Jul 27;8(7):nwaa173. doi: 10.1093/nsr/nwaa173 (PMC8310735; doi:10.1093/nsr/nwaa173)
Supplement: nwaa173_Supplemental_File [file nwaa173_supplemental_file.pdf]

Supplementary Materials for

Rapid drift of the Tethyan Himalaya terrane before two-stage India-Asia collision

Jie Yuan, Zhenyu Yang*, Chenglong Deng*, Wout Krijgsman, Xiumian Hu, Shihu Li, Zhongshan Shen, Huafeng Qin, Wei An, Huaiyu He, Lin Ding, Zhengtang Guo and Rixiang Zhu

*Corresponding authors. Emails: cldeng@mail.iggcas.ac.cn; zhenyu.yang@cnu.edu.cn.

This file includes:

- Supplementary Notes 1 to 4
- Supplementary Figs 1 to 14
- Supplementary Tables 1 to 4
- Supplementary References

Supplementary Note 1: Geologic settings

The Indus-Yalung Tsangpo Suture (Fig. 1b) represents the collision zone between the Lhasa and Tethyan Himalaya terranes, and thus between the Indian and Asian continents, during the early Cenozoic. It comprises the late Triassic–Eocene Gangdese magmatic arc, the early Cretaceous–Paleocene Xigaze forearc basin and the accretionary prism [1–4]. To the south, Ordovician–Eocene Tethyan Himalaya strata were deposited on the Indian continental passive margin, with shallow-water platform-facies deposits in the south (southern Tethyan Himalaya) to deep-water facies in the north (northern Tethyan Himalaya) [5].

In the Gyangze basin (Fig. 1c), Jurassic–Cretaceous strata were continuously deposited in a continental slope environment, sourced from the Indian continent [6–8]. The deep-water Cretaceous strata are subdivided into a lower (Gyabula Formation) and an upper (Chuangde Formation) unit [7]. The Gyabula Formation is composed of dark gray siliceous/calcareous shales, siltstones, and turbidite beds and is conformably overlain by the Chuangde Formation. The presence of fossil belemnites, ammonites, foraminifera and radiolarian in the Gyabula Formation indicates a lower continental slope depositional environment [7]. The Chuangde Formation of Santonian–Campanian age consists of CORBs, here represented as violet-red shales intercalated with thin marlstone beds [6,8,9]. Facies assemblages also suggest that the CORBs in the Cailangba section, that is located in the western part of the Gyangze area (Fig. 1c), were deposited in outer base-of-slope apron to deep basin environments [6]. The Cailangba B section is about 30 m thick and mainly consists of purple medium bedded limestones intercalated with conglomerates. The Cailangba A section is about 16 m thick, and comprises the same lithology as the lower-middle part of section B (Supplementary Fig. 2).

The Sangdanlin and Mubala sections in the Saga area (Fig. 1d), ~500 km west of the Gyangze region, represent the most distal continental margin of the northern Tethyan Himalaya [10–12]. The Paleogene strata in the Saga area are commonly divided into three formations [10–13] (Supplementary Fig. 3). The uppermost Cretaceous–Paleocene Denggang Formation consists of a lower interval of shales and siltstones, with radiolarian cherts and siliceous shales, and an upper interval of sandstones intercalated with cherts and shales. Upward the Paleocene–Eocene Sangdanlin Formation and Zheya Formation are composed of a shale/chert-dominated interval and an interval of sandstones interbedded with shales and tuffs [10–14]. The deep-water Sangdanlin Formation records the provenance change from Indian sources to Asian sources while the overlying Zheya Formation is dominantly sourced from the Gangdese arc to the north [10,13,14].

We sampled the upper Cretaceous Cailangba A and B sections and the Paleogene Sangdanlin and Mubala sections for paleomagnetic purposes, to reconstruct the plate tectonic history of the Tethyan Himalaya terrane by determining paleolatitudinal changes through time. The samples were first subjected to detailed scanning electron microscopy (SEM) observations and rock magnetic analyses (Fig. 2) to separate and verify the primary or secondary character of the different magnetization components (Fig. 3).

Supplementary Note 2: Anisotropy of magnetic susceptibility (AMS)

AMS measurements were performed using a KLY-4s Kappabridge. To avoid potential problems associated with heating, we completed the AMS measurements before any thermal demagnetization was conducted.

In stratigraphic coordinates, the maximum (k1) and mediate (k2) principle

anisotropy directions are parallel to the bedding with well grouped magnetic lineations; the minimum (k3) principle anisotropy directions are basically perpendicular to the bedding (Supplementary Fig. 9). These distributions suggest that the sedimentary magnetic fabrics of the samples of the Sangdanlin and Mubala sections have been subjected to incipient deformation [15]. Great-circle fitting the distribution of k3 of the Sangdanlin and Mubala sections provides planes with strike = 69.3°, dip = 88.3° (Supplementary Fig. 9b) and strike = 178.3°, dip = 89.2° (Supplementary Fig. 9d), respectively. We assume that the original stress pattern in both regions was the same with a north-south compression direction. Obviously a north-south compression direction inferred from the Mubala section is consistent with the regional tectonic context. We infer that a local counterclockwise rotation of 71° is required to fit the distribution of k3 of the Sangdanlin section with the north-south compression direction of the Mubala section. The characteristic of sedimentary magnetic fabrics of the samples in the Cailangba A and B sections is similar to that in the Sangdanlin and Mubala sections (Supplementary Figs. 9e, f).

Supplementary Note 3: Fold tests

The sample-mean direction is D_g = 163.8°, I_g = –20.4°, k = 2.7, α_{95} = 11.7°, N = 86 before tilt correction, and D_s = 176.3°, I_s = –18.8°, k = 35.8, α_{95} = 2.6° after tilt correction (Supplementary Fig. 10a, b). The significant increase of precise parameter k after tilt correction (k_s/k_g = 13.34 > F (170, 170) = 1.29) indicates a positive fold test at the 95% confidence limit [16]. In the fold test of McFadden [17], the calculated values are $\xi_{(2)\text{in situ}}$ = 81.56 in geographic coordinates and $\xi_{(2)\text{tilt corrected}}$ = 0.39 after tilt correction, while the critical value is ξ_C = 10.79 at 95% confidence limit, also indicating a positive fold test. Applying the progressive unfolding of Watson and

Enkin [18] shows a maximum precision parameter (k_{max} = 35.7) at 100% unfolding, and the direction (D_m = 356.2°, I_m = 18.7°, k = 35.7, α_{95} = 2.5°) is consistent with the mean direction after tilt correction (Supplementary Fig. 10).

Supplementary Note 4: Inclination shallowing test and correction

Five Cailangba hand samples from 5 sampling beddings oriented on the stratigraphic bedding plane were used for anisotropy-based inclination shallowing correction. A total of 23 specimens were drilled from the 5 hand samples in the direction perpendicular to the stratigraphic bedding. These specimens were subjected to successively increasing direct current (DC) fields (in the range of 0–1000 mT) applied at a 45° angel to bedding plane of the sampled strata [19]. In the process of applying the DC fields, IRM parallel (IRMx) and perpendicular (IRMz) to the bedding plane of each specimen were measured after each application of the DC field [19]. Then thermal demagnetization (in the temperature range of 100–680°C) was also conducted on these specimens (Supplementary Fig. 11). The values of IRMz/IRMx (IRM perpendicular/IRM parallel) of 23 specimens ranging of 200–1000 mT changed between 0.6273 and 0.7454, while those of IRMz/IRMx in the thermal demagnetization temperature interval of 650–690°C changed between 0.6176 and 0.7346, respectively (Supplementary Fig. 11 and Supplementary Table 2). Having considered that the HTCs were within 650–690°C, the mean IRMz/IRMx of each hand sample in the temperature range of 650–680°C was used to calculate the mean IRMz/IRMx of the sampled Cailangba section to be 0.6502.

The elongation/inclination (E/I) correction can be performed on the paleomagnetic data from the Cailangba B section (78 specimens), and from the Cailangba A and B sections (127 specimens), respectively (Supplementary Fig. 12).

The sample-mean direction of the Cailangba B section is D_s = 10.5°, I_s = –26.3°, k = 41.5, α_{95} = 2.5° after tilt correction. The corresponding E/I corrected inclination increased to –34.0°, with the best estimate between –26.9° and –43.7° at the 95% confidence level (Supplementary Fig. 12a). The sample-mean direction of the Cailangba A and B sections is D_s = 10.3°, I_s = –25.9°, k = 35.9, α_{95} = 2.1° after tilt correction. The corresponding E/I corrected inclination increased to –35.0°, with the best estimate between –29.5° and –39.7° at the 95% confidence level (Supplementary Fig. 12b).

Four Sangdanlin hand samples from 4 sampling bedding oriented on the stratigraphic bedding plane were used for anisotropy-based inclination shallowing correction. A total of 33 specimens were drilled from the 4 hand samples in the direction perpendicular to the stratigraphic bedding. These specimens were subjected to successively increasing DC fields (in the range of 0–800 mT) applied at a 45° angel to bedding plane of the sampled strata [19]. In the process of applying the DC fields, the IRMx and IRMz to the bedding plane of each specimen were measured after each application of the DC field [19]. Then thermal demagnetization (in the temperature range of 100–680°C) was also conducted on these specimens (Supplementary Fig. 13). The values of the IRMz/IRMx of 33 specimens in the range of 200–800 mT changed between 0.6924 and 1.0000, while those of IRMz/IRMx in the thermal demagnetization temperature interval of 650–690℃ changed between 0.5317 and 0.9827, respectively (Supplementary Fig. 13 and Supplementary Table 2). Having considered that the HTCs were determined within 650–690℃ by thermal demagnetization, the mean IRMz/IRMx of each hand sample in this temperature range was used to calculate the mean IRMz/IRMx of the section to be 0.6982.

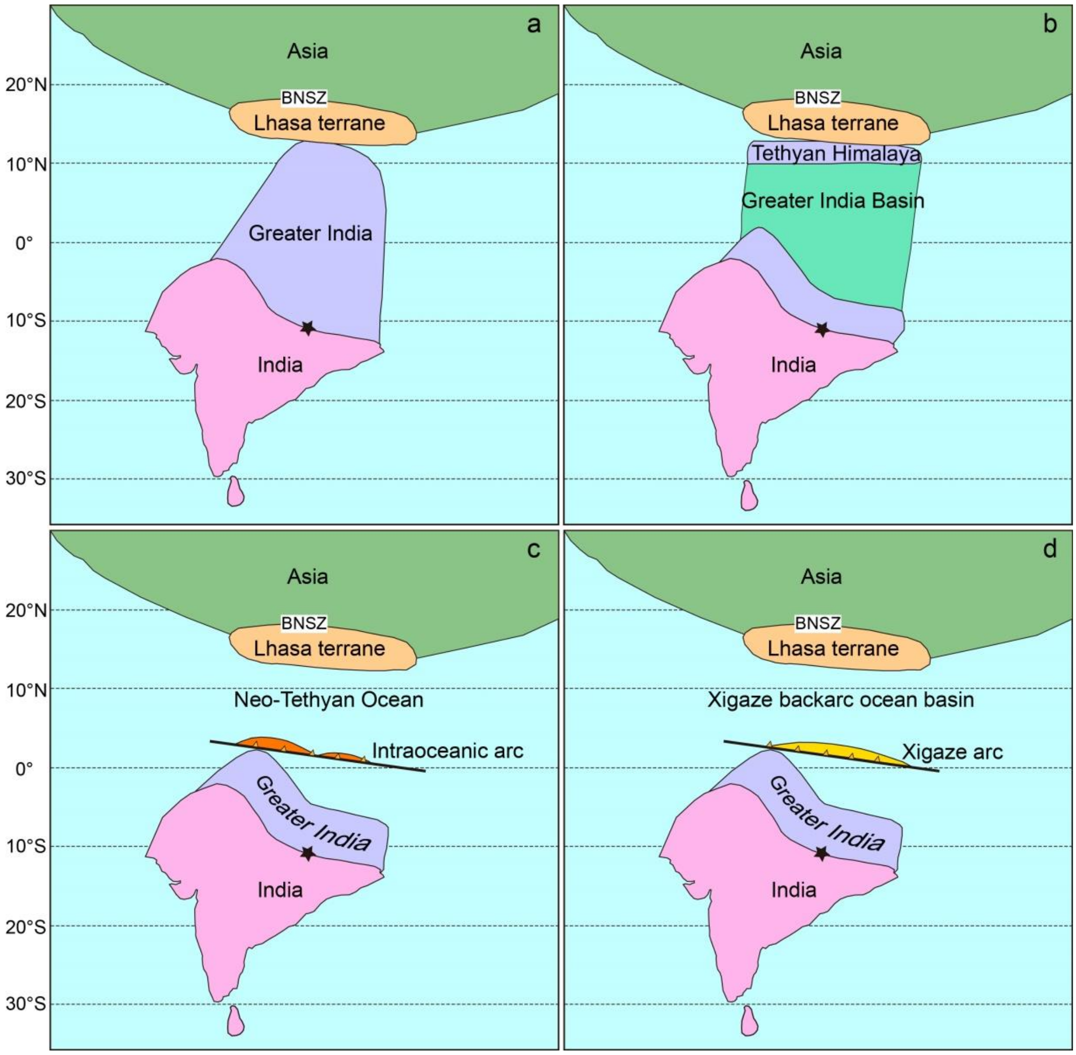

Supplementary Figure 1. Geodynamic models for reconstructions of India-Asia paleogeography at ~60 Ma. (a) The continental Greater India model [20,21]. (b) The Greater India Basin model [22,23]. (c) The island arc-continent collision model [24]. (d) The India-arc-Xigaze backarc ocean collision model [25].

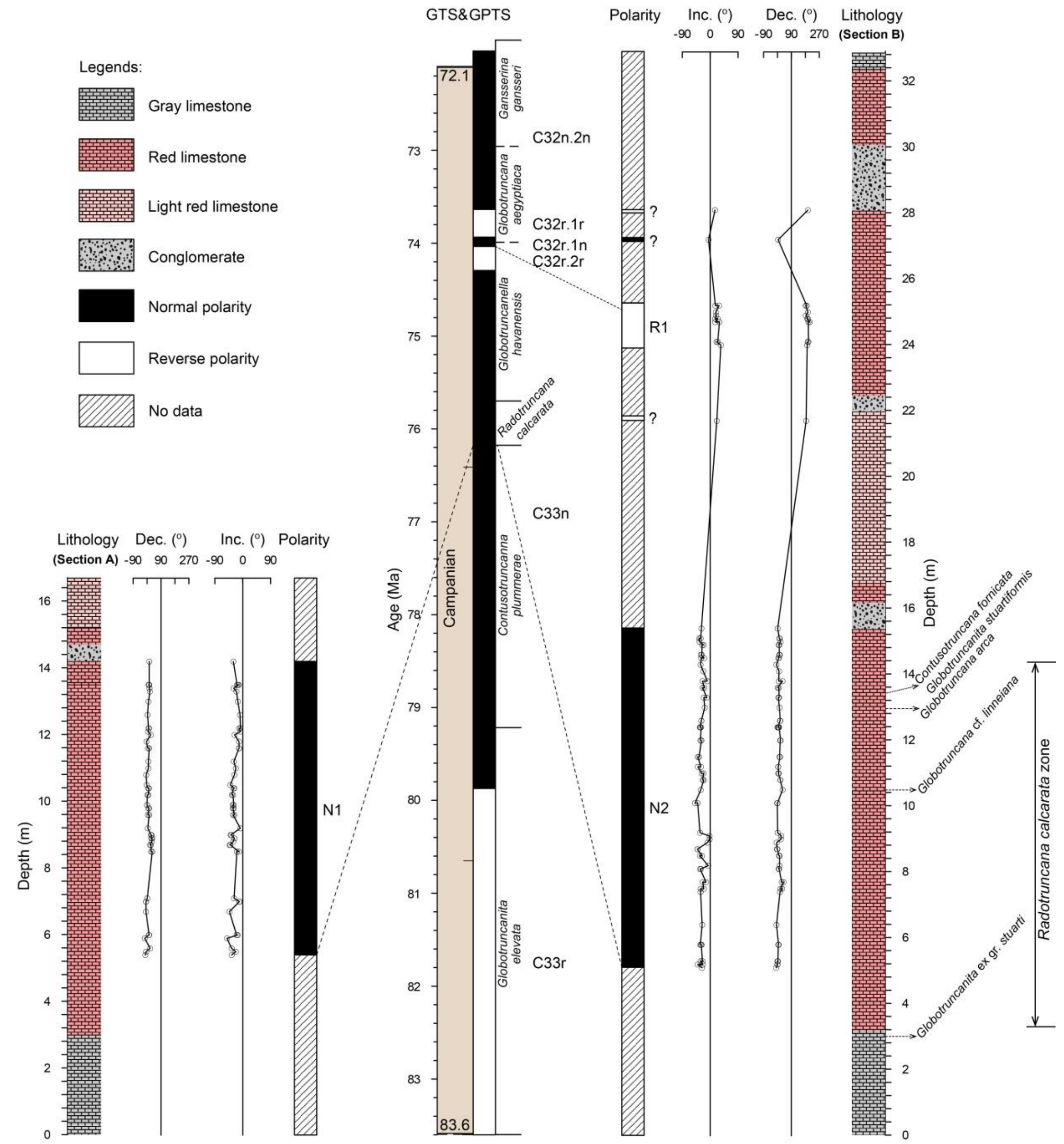

Supplementary Figure 2. Lithology, magnetostratigraphy and biostratigraphy of the Cailangba section. Dec., declinations; Inc., inclinations; GTS&GPTS, the geologic time scale and the geomagnetic polarity timescale [26]. Biostratigraphic data are after Chen *et al*. [6].

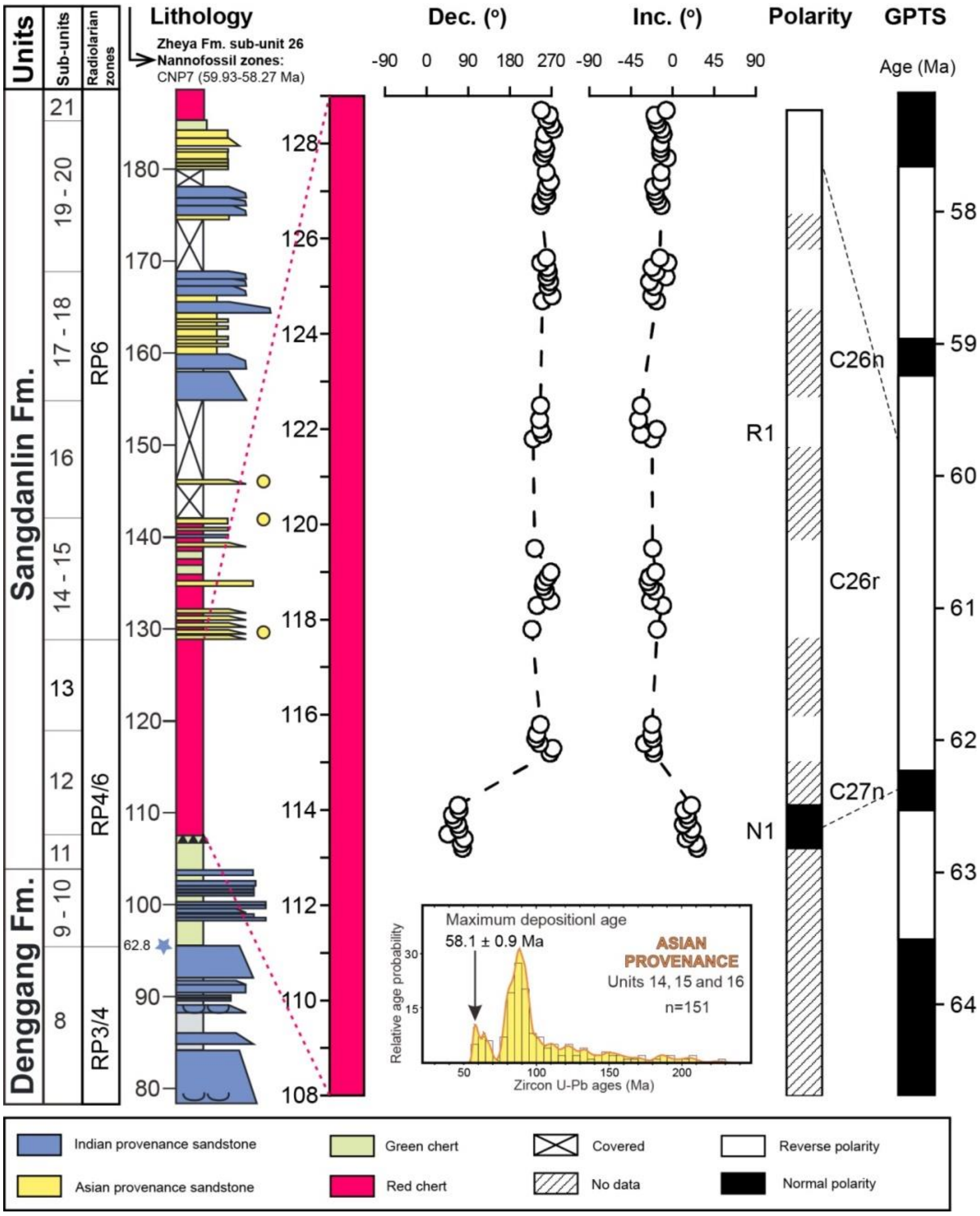

Supplementary Figure 3. Lithologic, biostratigraphic and magnetostratigraphic results of the Sangdanlin red siliceous shales with declinations and inclinations plotted as a function of stratigraphic level and showing the correlation with the GPTS [26]. The first arrival of Asian-derived turbidites is recorded in sub-units 14–16, the maximum depositional age of which is constrained at 58.1 ± 0.9 Ma by detrital zircon U-Pb geochronology [13].

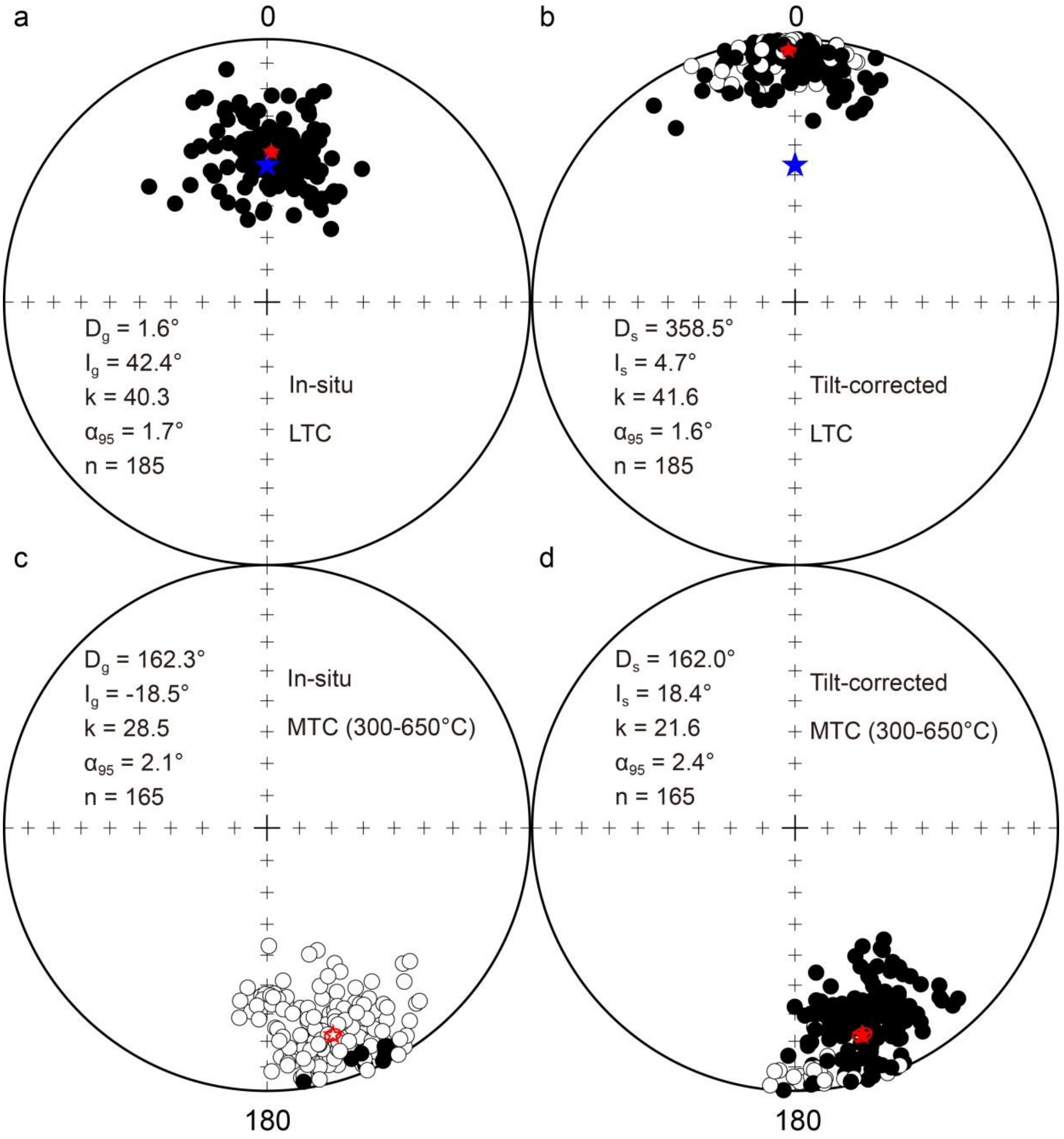

Supplementary Figure 4. Equal-area projections of in-situ (left) and tilt-corrected (right) paleomagnetic directions of all specimens in the Cailangba A and B sections. (a, b) LTC directions. (c, d) MTC directions. Red circles around the red stars in (a–d) denote the 95% confidence limit and mean directions. Solid and open symbols denote the lower and upper hemisphere, respectively. The blue stars in the (a, b) denote the present geomagnetic direction of the Cailangba section.

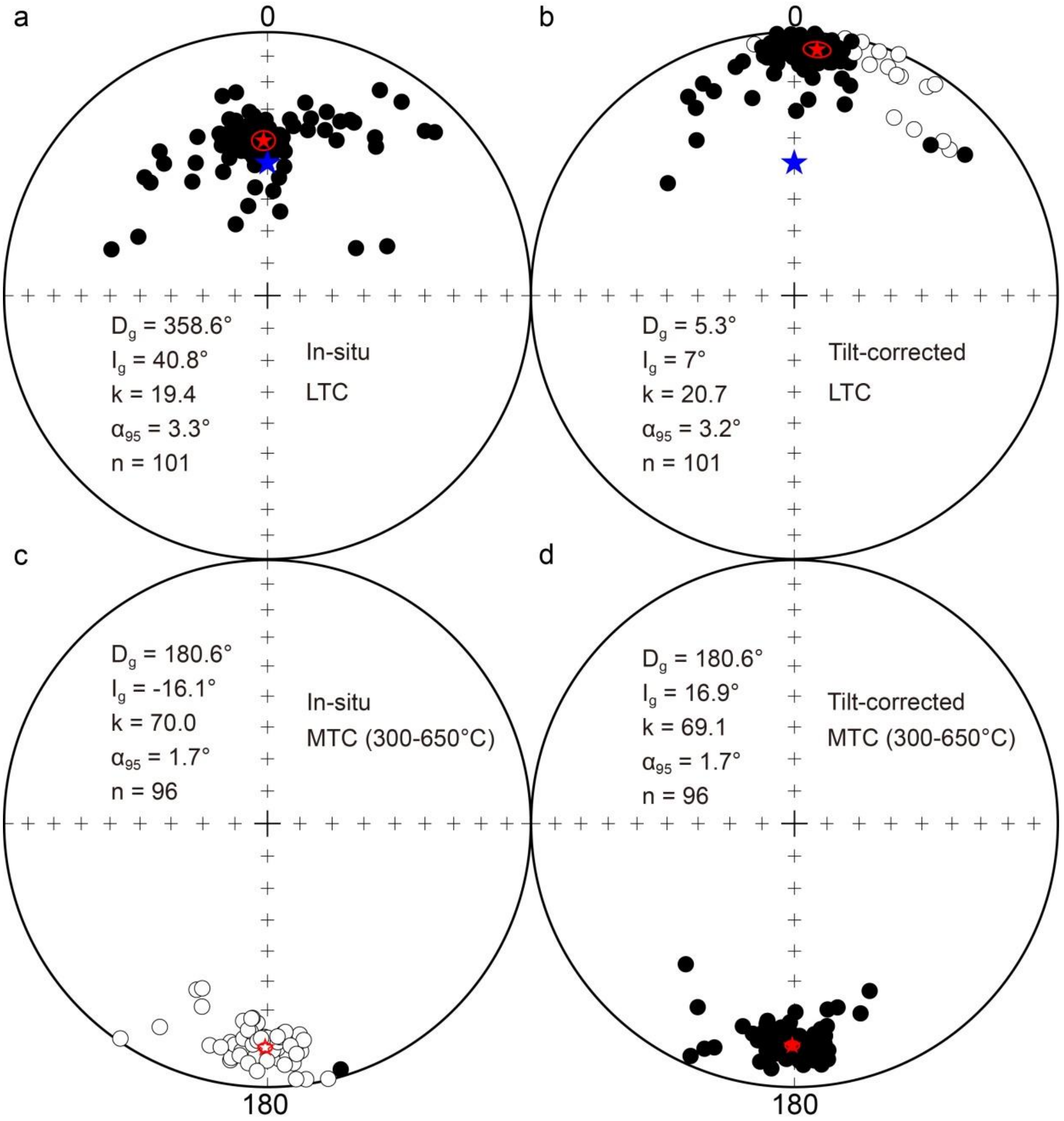

Supplementary Figure 5. Equal-area projections of in-situ (left) and tilt-corrected (right) paleomagnetic directions of all specimens in the Sangdanlin section. (a, b) LTC directions. (c, d) MTC directions. Red circles around the red stars in (a–d) denote the 95% confidence limit and mean directions. Solid and open symbols denote the lower and upper hemisphere, respectively. The blue stars in the (a, b) denote the present geomagnetic direction of the Sangdanlin section.

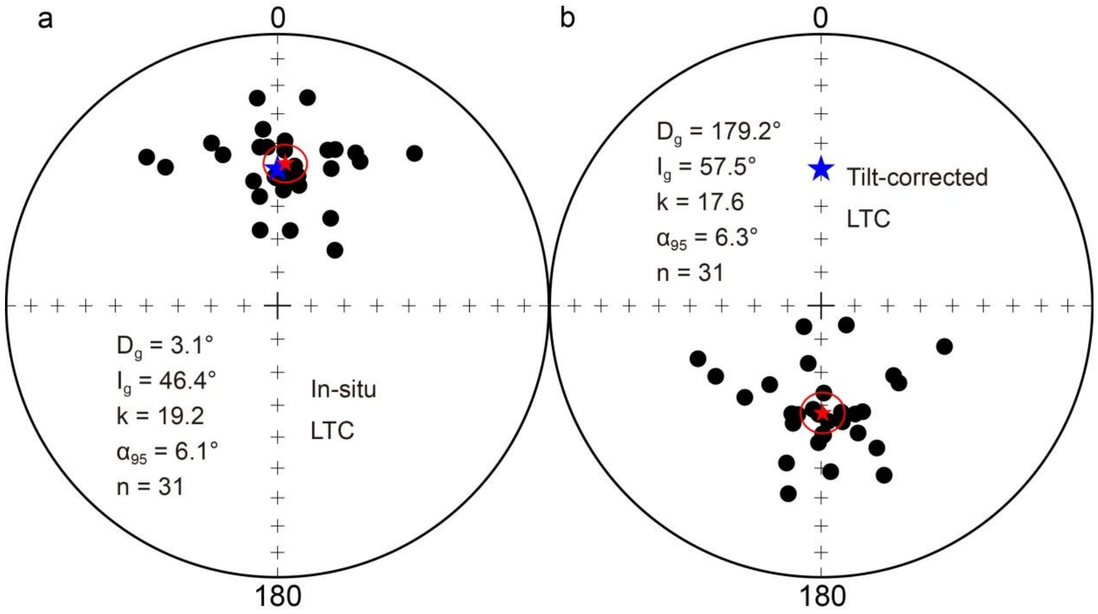

Supplementary Figure 6. Equal-area projections of in-situ (a) and tilt-corrected (b) LTC directions of all specimens in the Mubala section. Red circles around the red stars in (a, b) denote the 95% confidence limit and mean directions. Solid symbols denote the lower hemisphere. The blue stars in the (a, b) denote the present geomagnetic direction of the Mubala section.

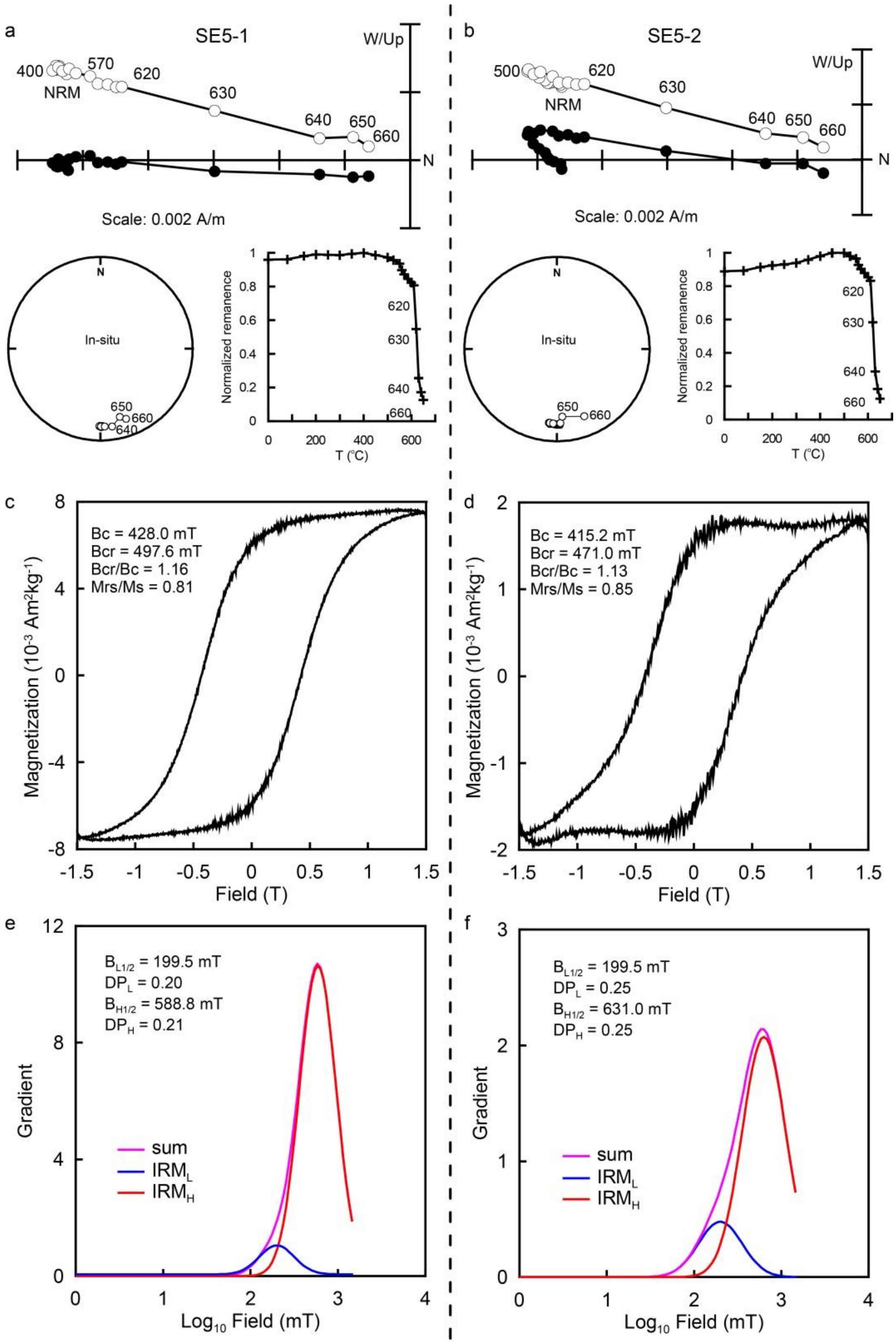

Supplementary Figure 7. Paleomagnetic and rock magnetic results of the completely remagnetized specimens of Sangdanlin section. (a, b) Representative orthogonal demagnetization diagrams in geographic coordinates, corresponding normalized NRM vs. temperature plots, equal-area stereonets. (c, d) Hysteresis loops after high-field slope correction with hysteresis parameters indicated. (e, f) IRM component analysis, where blue, red and purple lines indicate the low (IRM_L), high coercivity (IRM_H) components and the sum of these components (sum), respectively.

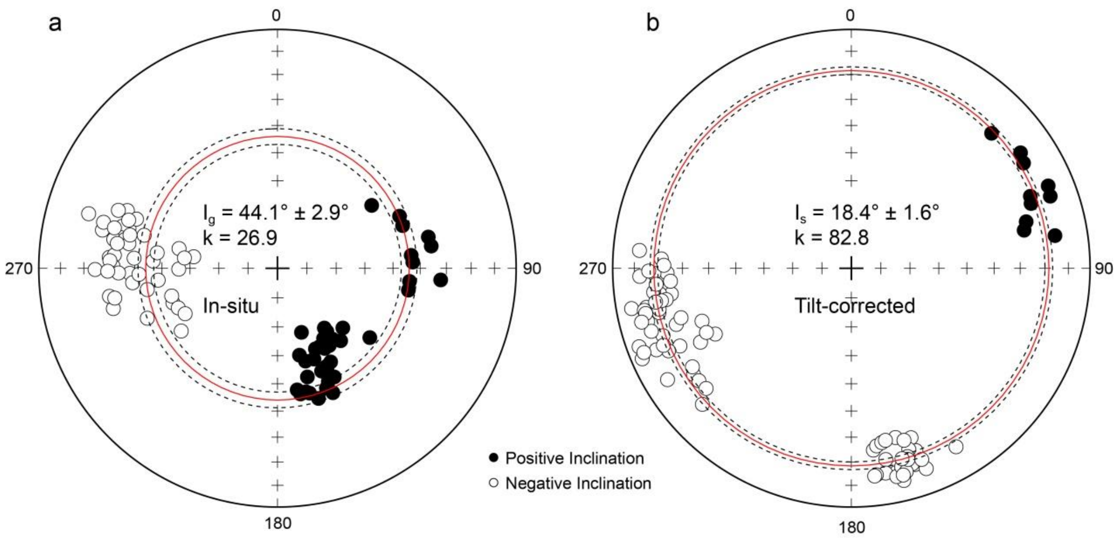

Supplementary Figure 8. Equal-area projections of sample-mean direction of HTCs for the Sangdanlin and Mubala sections. (a) Before tilt correction. (b) After tilt correction. The red circles with black dashed circles show the average inclination and error of the average inclination.

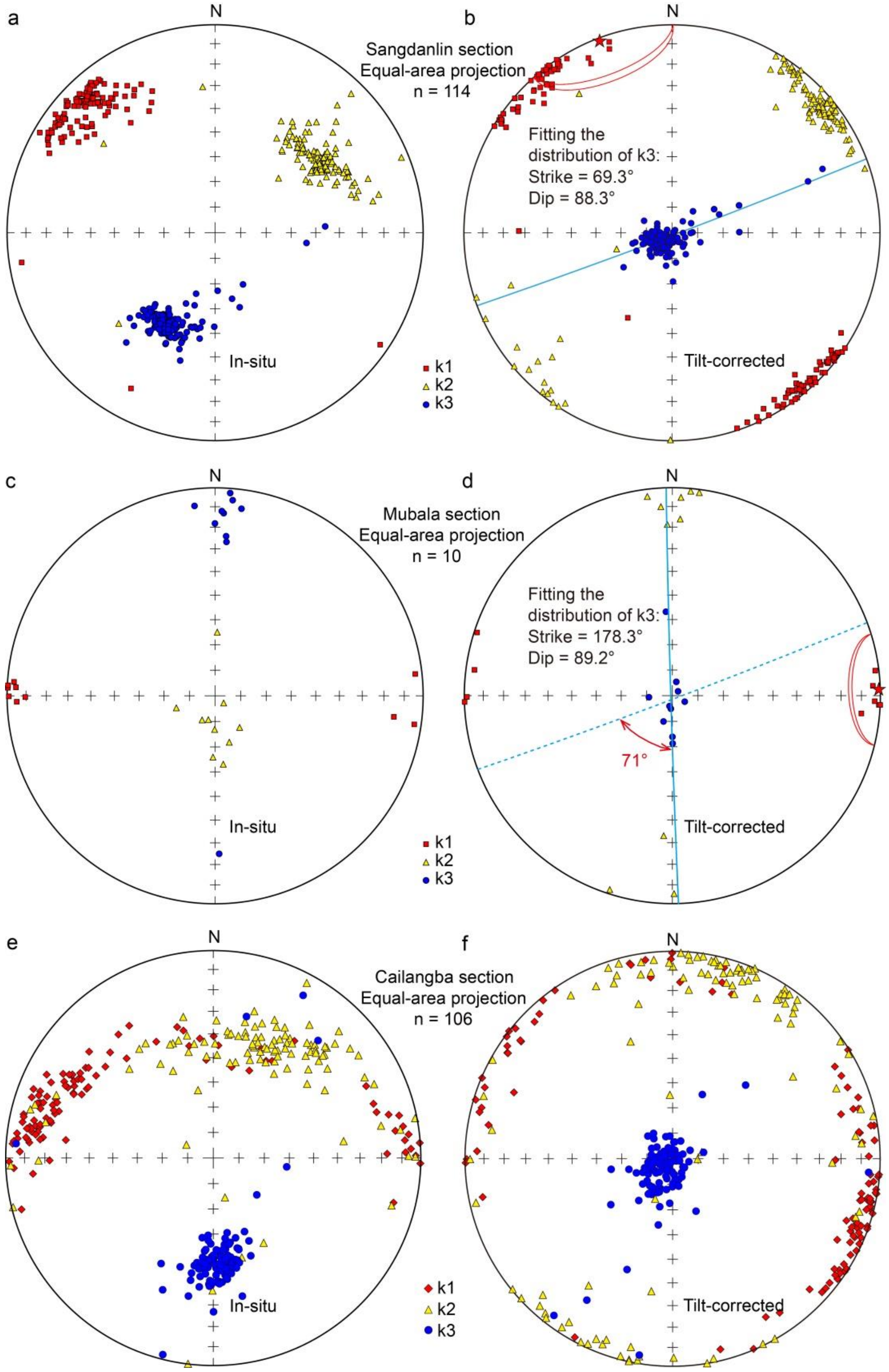

Supplementary Figure 9. Stereoplots of the AMS results of the Sangdanlin (a, b), Mubala (c, d) and Cailangba (c, d) sections in geographical (stratigraphic) coordinates. Solid symbols, individual directions. Light blue line, the plane of great-circle fitting the distribution of k3. Red star, the pole to plane of fitting the distribution of k3 with 95% confidence limit.

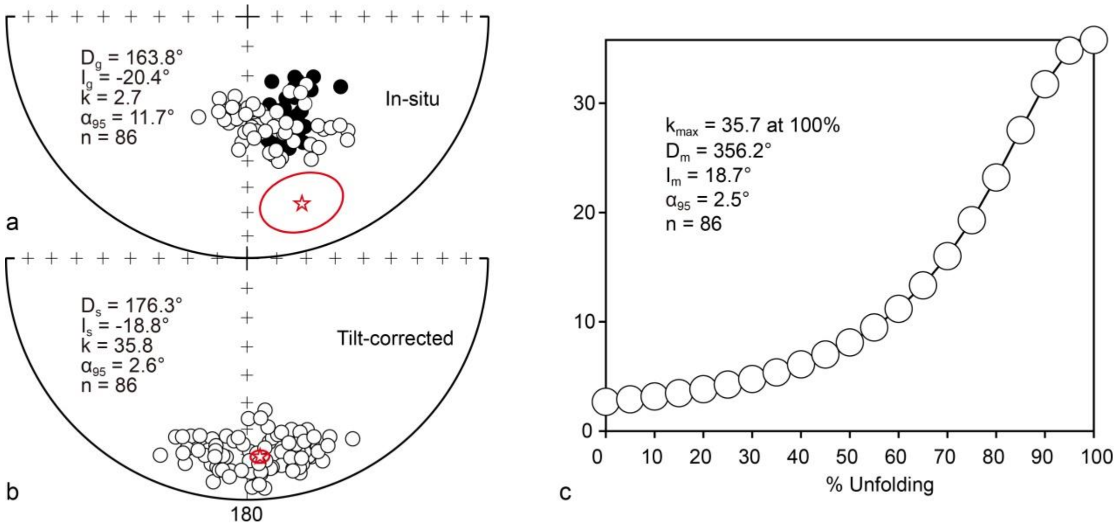

Supplementary Figure 10. Equal-area projections of in-situ (a) and tilt-corrected (b) paleomagnetic directions and progressive unfolding of the mean direction (c) of Sangdanlin section after rotated 71 ° counterclockwise and of Mubala section. Red circles around the red stars in (a, b) denote the 95% confidence limit and mean directions. Solid and open symbols in (a, b) denote the lower and upper hemisphere, respectively.

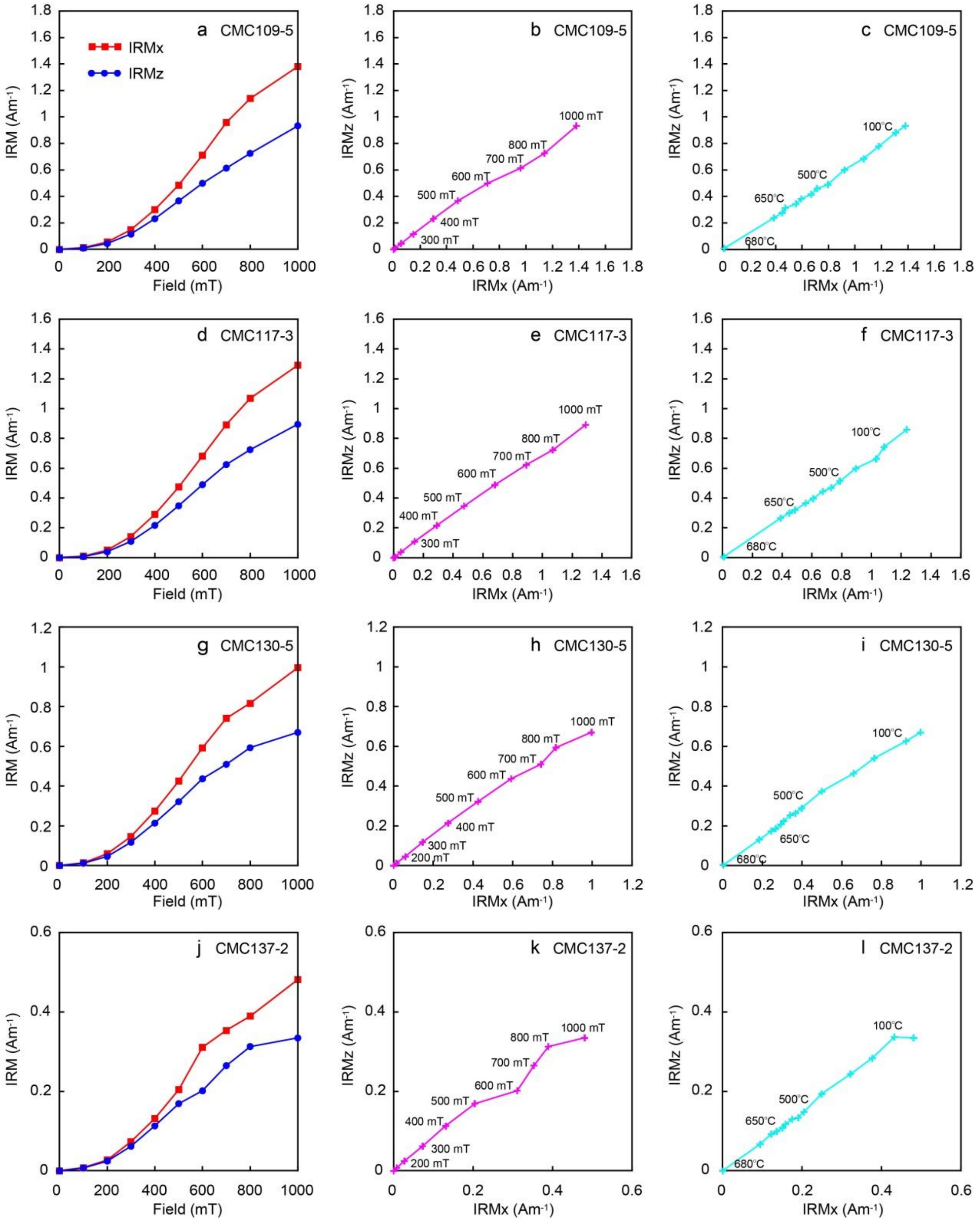

Supplementary Figure 11. The anisotropy-based inclination shallowing correction for the CORBs of the Cailangba section. (a, d, g and j) The IRMx (parallel to bedding) and IRMz (perpendicular to bedding) acquisitions produced by applying a magnetic field at an angle of 45 °to the bedding as a function of increasing field. (b, e, h and k) The slope (IRMz/IRMx) of the least-squares-fit for the data points between 200 mT and 1000 mT. (c, f, i and l) The slope of the thermal demagnetization of IRMz and IRMx, respectively.

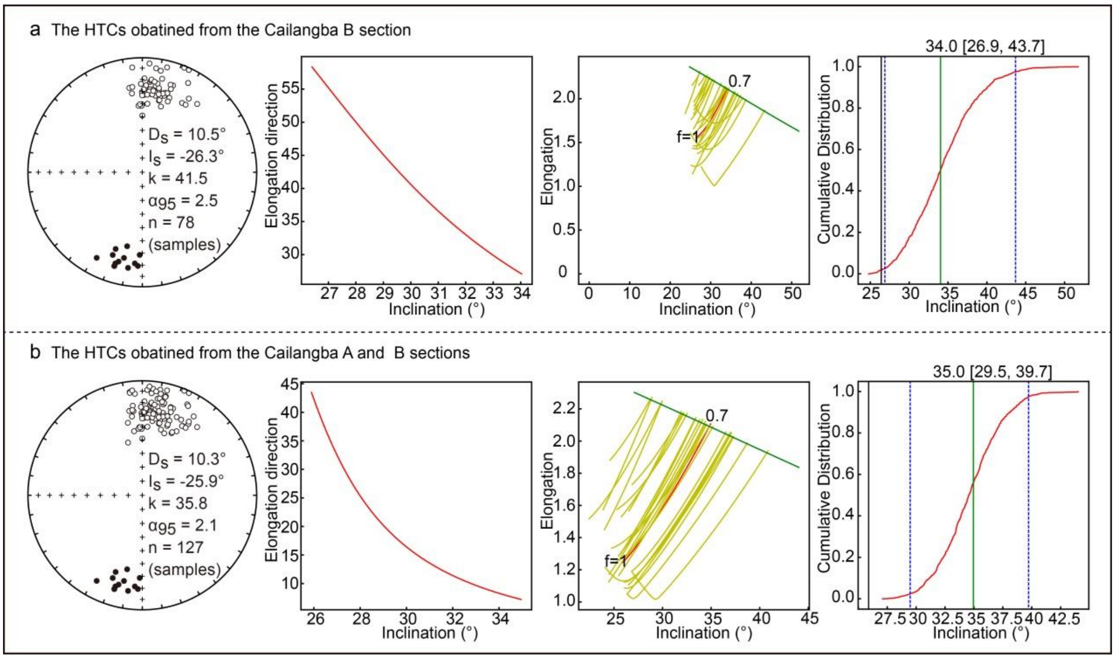

Supplementary Figure 12. The E/I inclination shallowing correction. (a) The data from the Cailangba B section. (b) The data from the Cailangba A and B sections. From left to right in (a, b) are the equal-area projections of samples directions, elongation direction of the curve with respect to inclination, elongation/inclination as a function and cumulative distribution of the corrected inclination.

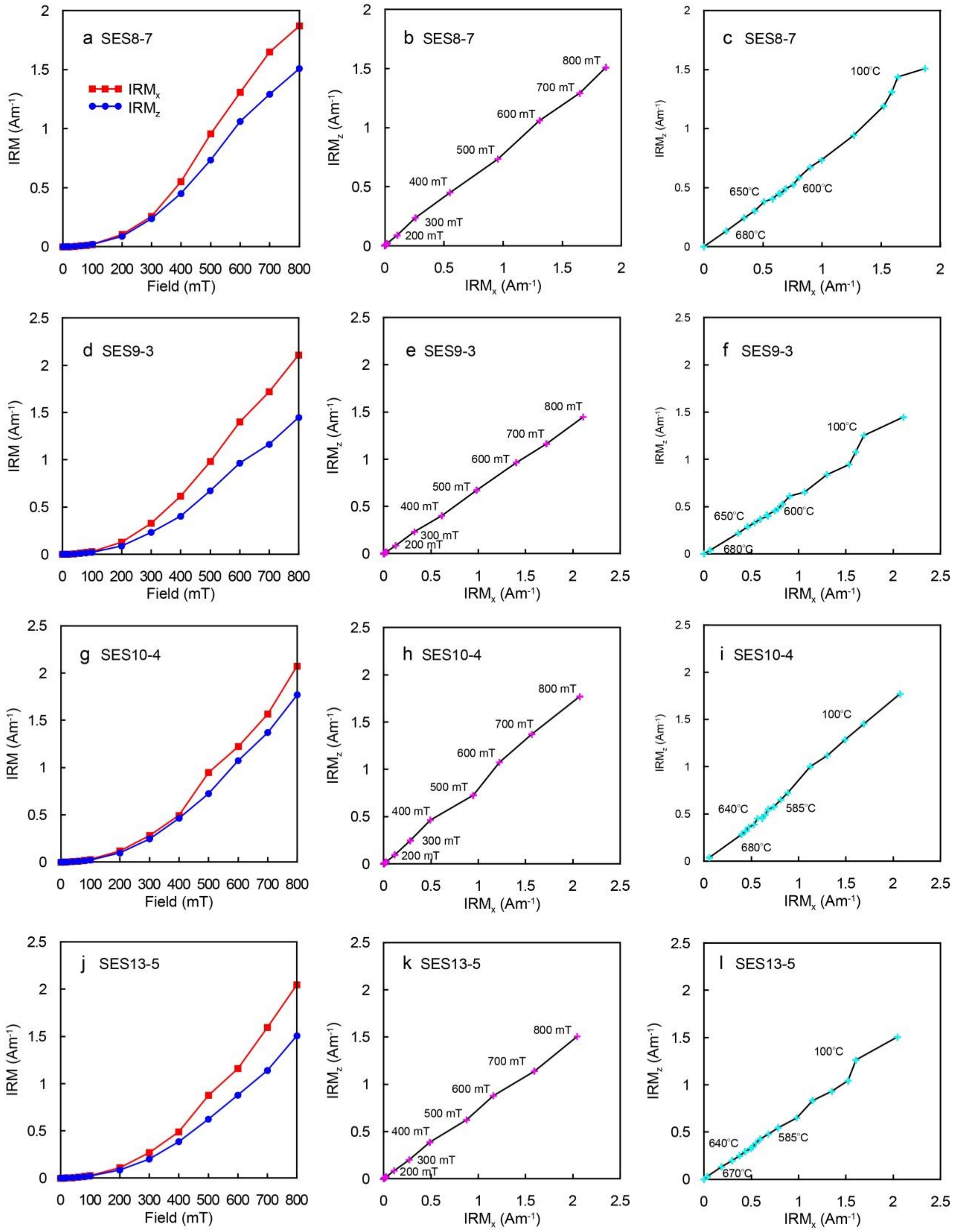

Supplementary Figure 13. The inclination shallowing correction for the Sangdanlin red siliceous shales. (a, d, g and j) The IRMx (parallel to bedding) and IRMz (perpendicular to bedding) acquisitions produced by applying a magnetic field at an angle of 45 ° to the bedding as a function of increasing field. (b, e, h and k) The slope (IRMz/IRMx) of the least-squares-fit for the data points between 200 mT and 800 mT. (c, f, i and l) The slope of the thermal demagnetization of IRMz and IRMx, respectively.

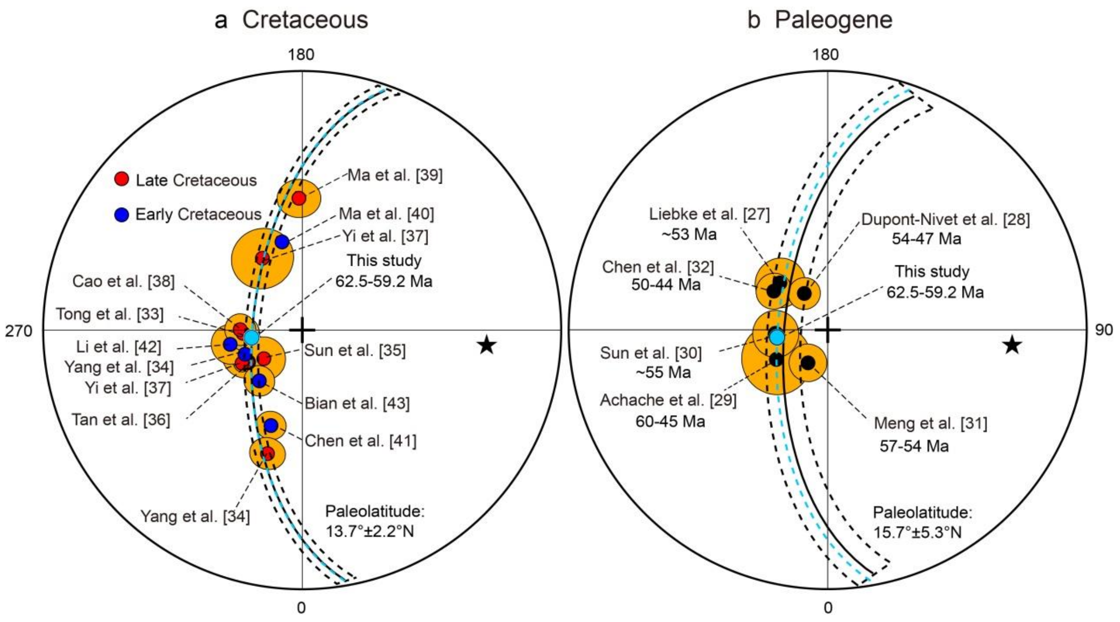

Supplementary Figure 14. Paleopoles of Cretaceous and Paleogene for the Lhasa terrane. (a) Equal-area projections showing the distribution of Cretaceous paleopoles. Small-circle fitting passes through 13 selected Cretaceous poles, and gives a paleolatitude of 13.7° ± 2.2°N. (b) Equal-area projection of the Paleogene paleopoles. Small-circle fitting passes through 6 selected Paleogene poles, indicating a paleolatitude of 15.7° ± 5.3°N. See Supplementary Table 4 for values and sources. The black star indicates the sampling location, which was selected as the reference point (29.3°N, 85.3°E) when calculating the paleolatitude of the Lhasa terrane in this study.

Supplementary Table 1. High-temperature component directions of all specimens in the Sangdanlin, Mubala, Cailangba A and B sections.

Sample ID	Bedding Strike/dip	Depth (m)	D_g (°)	I_g (°)	D_s (°)	I_s (°)	MAD (°)	Demag
**Sangdanlin section (650-680 ℃)**								
SE6-1	297/34	113.2	99.5	43.7	77.6	26.8	9.4	Th
SE6-2	297/34	113.3	95.7	43.7	75.1	25.2	2.2	Th
SE6-3	297/34	113.4	94.1	32	80.9	14.7	7.5	Th
SE6-4	297/34	113.5	56.3	50.8	46.1	19.6	3.4	Th
SE6-5	297/34	113.6	87.4	42.7	70.2	21.1	5.1	Th
SE6-6	297/34	113.7	78.3	35.7	67.3	11.6	8	Th
SE6-7	297/34	113.8	71.2	43.9	58.6	16.6	2.9	Th
SE6-8	297/34	113.9	66.9	43.7	55.7	15.3	3	Th
SE6-9	297/34	114	81.8	35.2	70.1	12.4	5.5	Th
SE6-10	297/34	114.1	84.5	43.1	68.1	20.3	19.3	Th
SE8-1	300/36	115.2	285.1	-35.8	266.8	-20.5	5.2	Th
SE8-2	300/36	115.3	291	-33.1	272.8	-21.4	18.3	Th
SE8-3	300/36	115.4	269.5	-56	243.9	-30.3	9.1	Th
SE8-4	300/36	115.5	251.7	-51.9	236.1	-21.4	6.2	Th
SE8-5	300/36	115.6	255.3	-52.2	238.2	-22.7	6.1	Th
SE8-7	300/36	115.8	264.3	-48.4	245.6	-22.2	5.8	Th
SE9-2	300/36	117.8	236.9	-50.2	227.6	-16.6	8.2	Th
SE9-7	300/36	118.3	249	-41.2	238.8	-10.9	0.4	Th
SE9-8	300/36	118.4	289.2	-37.5	268.5	-23.8	2.9	Th
SE9-10	300/36	118.6	274	-39.6	257	-18.4	3.4	Th
SE9-11	300/36	118.7	273.3	-46.7	252.2	-24	3.6	Th
SE9-12	300/36	118.8	277.3	-47.9	253.8	-26.6	4.3	Th
SE9-13	300/36	118.9	283.9	-42	261.9	-24.8	11.2	Th
SE9-14	300/36	119	285.1	-33.1	268.5	-18.4	13.9	Th
SE9-19	300/36	119.5	248.2	-53.1	233.6	-21.7	2.1	Th
SE10-2	300/36	121.8	243.8	-54.5	230.4	-22.1	4.8	Th
SE10-3	300/36	121.9	281.3	-55.4	250.5	-34	9.3	Th
SE10-4	300/36	122	260.7	-44	245.7	-17.1	4.4	Th
SE10-6	300/36	122.2	277.8	-61	244.2	-36.8	8.7	Th
SE10-9	300/36	122.5	276.3	-57.8	246.1	-34	10.2	Th
SE11-1	291/38	124.7	268	-38.1	250.3	-17.3	3.2	Th
SE11-2	291/38	124.8	290	-29.8	270.9	-22.5	2.4	Th
SE11-4	291/38	125	281.1	-33.2	262.4	-20.1	3.6	Th
SE11-5	291/38	125.1	287.9	-34.8	265.9	-25	3.7	Th
SE11-6	291/38	125.2	269.6	-24.6	259.6	-7.1	2.4	Th
SE11-7	291/38	125.3	280	-31	263.1	-17.7	3.5	Th
SE11-8	291/38	125.4	281.3	-36.2	260.4	-22.4	2.7	Th
SE11-9	291/38	125.5	256.2	-29.9	246.6	-5.1	14.9	Th
SE11-10	291/38	125.6	273.7	-30.1	259.2	-13.7	5.4	Th
SE12-1	293/42	126.7	264.3	-38.9	247.3	-12.5	3.5	Th
SE12-2	293/42	126.8	267.5	-41.5	247.7	-16	2.5	Th
SE12-3	293/42	126.9	280.7	-35.3	260.1	-18.3	2.4	Th
SE12-4	293/42	127	279.4	-37	258	-18.8	2.7	Th
SE12-5	293/42	127.1	282.7	-36.8	260.3	-20.4	2.1	Th
SE12-6	293/42	127.2	282.3	-25.9	267.8	-12.3	11.2	Th

SE12-8	293/42	127.4	274.6	-32	258.5	-12.5	3.2	Th
SE13-1	290/40	127.7	260.5	-29.4	249.7	-5.8	2.9	Th
SE13-2	290/40	127.8	270.7	-31.7	255.4	-12.8	3.1	Th
SE13-3	290/40	127.9	272.6	-30.4	257.6	-12.8	3.7	Th
SE13-4	290/40	128	268.3	-33.8	252.5	-13.2	1.3	Th
SE13-6	290/40	128.2	269	-29.6	255.6	-10.2	1.1	Th
SE13-7	290/40	128.3	286.1	-18.8	274.8	-11.9	4	Th
SE13-8	290/40	128.4	284.8	-25.6	269.2	-16.2	2.1	Th
SE13-9	290/40	128.5	278.8	-32.6	260.2	-17.9	1.7	Th
SE13-10	290/40	128.6	283.9	-30.3	265.3	-19.1	5.8	Th
SE13-11	290/40	128.7	259.6	-31.3	247.9	-6.9	2.6	Th
Mubala section (600-680 ℃)								
MB1-4	90/72	-	170.8	47.2	173.1	-24.3	7.7	Th
MB1-5	90/72	-	164.6	51	169.8	-19.7	5.4	Th
MB1-6	90/72	-	162.5	42.3	165.4	-27.6	3.1	Th
MB1-7	90/72	-	169.6	45.5	171.9	-25.8	10	Th
MB2-1	90/72	-	152.1	59.4	166.1	-9.3	7.7	Th
MB2-2	90/72	-	152.4	47.4	160.5	-20.1	7.2	Th
MB2-3	90/72	-	156	42.4	160.6	-25.7	6.4	Th
MB2-4	90/72	-	153.9	51.6	163.4	-16.7	7.7	Th
MB2-5	90/72	-	155.8	46.9	162.4	-21.5	4.6	Th
MB2-6	90/72	-	158.1	56.3	167.7	-13.4	7	Th
MB2-7	90/72	-	156.8	51.3	165	-17.8	6.4	Th
MB2-8	90/72	-	163.2	56.6	170.5	-14.1	8	Th
MB2-9	90/72	-	150.4	52.8	162	-14.7	4	Th
MB3-1	91/74	-	153.9	51.1	163.4	-18.8	3.3	Th
MB3-2	91/74	-	157.8	44.9	163	-25.6	6.8	Th
MB3-3	91/74	-	154.8	59.4	167.7	-11.7	2.6	Th
MB3-4	91/74	-	165.8	59.2	173.1	-13.9	3.4	Th
MB3-6	91/74	-	167	45.9	170.1	-26.8	3.5	Th
MB3-7	91/74	-	165.6	45.1	168.8	-27.4	1.3	Th
MB3-9	91/74	-	153.3	52.1	163.6	-17.7	1.4	Th
MB4-1	93/82	-	146.7	61.2	165.8	-15.3	7.2	Th
MB4-2	93/82	-	142.2	59.2	162.7	-15.3	4.9	Th
MB4-3	93/82	-	132.4	59.6	159.5	-11.4	3	Th
MB4-4	93/82	-	142	62.5	164.9	-12.8	3.2	Th
MB4-5	93/82	-	138.9	56.9	159.8	-15.8	2.5	Th
MB4-8	93/82	-	126.9	50	149.6	-14.4	2.7	Th
MB4-9	93/82	-	159.5	66.6	173.6	-13.5	2.4	Th
MB4-10	93/82	-	141.9	64	165.9	-11.7	2.8	Th
MB4-11	93/82	-	146.2	58	163.5	-17.6	2.5	Th
MB4-12	93/82	-	149.5	58	165.1	-18.6	2.1	Th
**Cailangba A section (650-680 ℃)**								
CL025	261/33	5.4	349.2	-2.7	348.7	-35.7	0.8	Th
CL026	261/33	5.5	353.7	10.6	353.9	-22.4	2.5	Th
CL027	261/33	5.6	14.1	-6	19.7	-35.9	1.8	Th
CL028	261/33	5.9	346.9	-17.8	344.8	-50.6	1	Th
CL029-1	261/33	6	11.4	14.4	11.6	-16.6	7.6	Th
CL029-2	261/33	6	12.1	11.2	13.1	-19.6	4.7	Th

CL032-1	261/33	6.7	352.2	-10	352.7	-43	2.4	Th
CL033-1	261/33	7	353.1	22.9	353	-10.1	1	Th
CL033-2	261/33	7	353.1	22.2	353	-10.8	0.9	Th
CL034	261/33	7.1	0.1	4.6	1.3	-27.9	2.2	Th
CL040-1	268/36	8.5	31.7	11.1	33.1	-18.9	4.9	Th
CL040-2	268/36	8.5	27.4	19.4	26.3	-12.4	4.1	Th
CL041-1	268/36	8.7	18.7	-6.7	25.2	-39.8	3.4	Th
CL041-2	268/36	8.7	13.9	-7.8	19.5	-42.1	2.5	Th
CL042	268/36	8.8	25.3	1	30.2	-30.6	2.3	Th
CL043-1	268/36	8.9	28.8	3.8	32.9	-26.7	2.2	Th
CL043-2	268/36	8.9	23.9	5.4	27.2	-26.7	1.8	Th
CL044-1	268/36	9	21.9	-4.4	28.2	-36.7	3.1	Th
CL044-2	268/36	9	19	-5.9	25.3	-38.9	5.5	Th
CL046	268/36	9.2	5.5	28	4.7	-7.7	1.5	Th
CL047-1	268/36	9.6	11.1	3.8	13.3	-31.2	2.3	Th
CL047-2	268/36	9.6	4.7	8.8	5.4	-26.9	1.7	Th
CL048-1	269/40	9.8	10.4	7.5	12.3	-31.6	2.9	Th
CL048-2	269/40	9.8	8.2	10.3	9.4	-29.2	4.1	Th
CL049-1	269/40	9.9	359.9	7.7	0.1	-32.3	2.5	Th
CL049-2	269/40	9.9	359.4	11.1	359.5	-28.9	4.2	Th
CL050-1	269/40	10.2	1.7	8.7	2.1	-31.3	1.7	Th
CL050-2	269/40	10.2	6.2	4.8	7.7	-34.9	2.3	Th
CL051-1	269/40	10.4	9.7	9.1	11.2	-30.1	2.4	Th
CL051-2	269/40	10.4	6.3	12.5	7.1	-27.2	2.8	Th
CL052	269/40	10.5	357.9	-0.2	357.6	-40.2	4.9	Th
CL053	269/40	10.8	354.4	12.9	354	-27	2.8	Th
CL054	269/40	11	8.4	17.2	8.7	-22.3	3.2	Th
CL055	258/39	11.2	6.3	8.4	8.7	-28.6	5.7	Th
CL056-1	258/39	11.6	15.1	23.1	13.4	-12.2	2.9	Th
CL056-2	258/39	11.6	6.7	25.7	5.2	-11.5	2.4	Th
CL057	258/39	11.8	356.5	26.5	355.8	-12.1	1	Th
CL058	258/39	12	19.8	6.5	23.8	-26.3	4	Th
CL059	258/39	12.1	8.4	29.7	5.8	-7.3	1.1	Th
CL060-1	258/39	12.2	13.6	23.7	11.9	-11.9	3.1	Th
CL060-2	258/39	12.2	12.7	28.9	9.7	-7.2	1.5	Th
CL061	258/39	12.6	4.7	29	2.7	-8.7	1.5	Th
CL062	258/39	13	9.6	20.8	9	-15.7	1.3	Th
CL063-1	258/39	13.3	16.8	14.1	17.9	-20.2	2.4	Th
CL064	258/39	13.4	12.1	6	15.7	-29.3	2.5	Th
CL065-1	258/39	13.5	14.7	22.5	13.2	-12.9	1.6	Th
CL065-2	258/39	13.5	11	21.1	10.2	-15.1	3.3	Th
CL065-3	258/39	13.5	13.3	17.4	13.4	-18.2	1.5	Th
CL067	258/39	14.2	11.4	9	14.1	-26.7	2.7	Th
**Cailangba B section (650-680 ℃)**								
CM096-2	258/44	5.1	351	17.5	351.2	-26.4	0.9	Th
CM097-1	258/44	5.2	357.1	2.9	0	-40.5	0.6	Th
CM097-2	258/44	5.2	358.7	19.5	359.1	-23.8	5.3	Th
CM098-1	258/44	5.3	358.3	12.5	359.7	-30.7	9.4	Th
CM098-2	258/44	5.3	1.8	17.7	2.5	-25	2.7	Th
CM099-1	258/44	5.8	1.9	11.1	5	-29	3	Th

CM099-2	258/44	5.8	4.9	13.1	6.9	-29	4.1	Th
CM099-3	258/44	5.8	3.8	14.3	5.4	-28	3.7	Th
CM099-4	258/44	5.8	4.1	10.2	6.7	-31.9	3.6	Th
CM101-2	258/38	6.4	352.9	11.8	353.3	-26	4.5	Th
CM102	258/38	7.4	11.8	3.4	16	-31.1	2.6	Th
CM103-1	258/38	7.5	23	-0.6	29.9	-30.9	2	Th
CM103-2	258/38	7.5	18	12.2	19.5	-20.7	5.1	Th
CM104-1	258/38	7.7	32.9	1.6	38.8	-24.5	7.2	Th
CM104-2	258/38	7.7	27.8	16.3	27.2	-13.4	2.5	Th
CM105-1	258/38	8.1	6.1	3.2	9.7	-32.7	2.2	Th
CM105-2	258/38	8.1	4.8	6	7.4	-30.2	1.8	Th
CM106-2	258/38	8.2	16.5	27.6	13.3	-6.6	8.5	Th
CM108-1	258/38	8.5	8.9	1.9	13.3	-33.2	2.7	Th
CM108-2	258/38	8.5	7.1	8.8	9.3	-27	1.4	Th
CM109-1	258/38	8.7	356.5	-4.2	359.3	-41.6	1.8	Th
CM109-2	258/38	8.7	354.5	-3.7	356.7	-41.4	2.1	Th
CM110	258/38	8.9	355.3	28.8	354.5	-8.9	8.6	Th
CM111-2	258/38	9	27.7	29.1	21.9	-1.8	7.8	Th
CM112-2	258/38	9.1	30.1	27.8	24.5	-2.1	3.7	Th
CM113-2	258/38	9.2	358.9	4.2	1	-33	2.4	Th
CM117-1	263/43	10.1	356.9	2.6	358.1	-40.3	1.6	Th
CM117-2	263/43	10.1	357.6	-6.2	360	-49	1.1	Th
CM118	263/43	10.5	26.5	4.6	32.7	-30.6	2.4	Th
CM119-1	263/43	10.8	17.5	18.5	17.9	-20.8	2.2	Th
CM119-2	263/43	10.8	17.4	15.1	19	-24.2	2.2	Th
CM120-1	263/43	11	4.7	20.3	4.9	-21.8	3.1	Th
CM120-2	263/43	11	6.5	14.1	7.8	-27.7	2.4	Th
CM121-1	263/43	11.2	1.7	2.7	4.3	-39.8	2.5	Th
CM121-2	263/43	11.2	3.4	12.3	4.8	-30	4	Th
CM122-1	263/43	11.5	10.3	0.5	15.8	-40.1	3.6	Th
CM122-2	263/43	11.5	9.2	5.1	13.1	-36	3.5	Th
CM123-1	263/43	12	14.9	11.3	17.6	-28.4	2.8	Th
CM123-2	263/43	12	16	7.9	20	-31.4	3	Th
CM124-1	262/39	12.4	7.7	8.1	10	-29.3	1.5	Th
CM124-2	262/39	12.4	358.6	5.4	359.9	-33.3	2.7	Th
CM124-3	262/39	12.4	5	5.9	7.3	-32	4	Th
CM124-4	262/39	12.4	1.8	5.8	3.6	-32.6	3	Th
CM125-1	262/39	12.6	13	7.7	15.9	-28.6	5	Th
CM125-2	262/39	12.6	14.5	7.4	17.6	-28.4	3.7	Th
CM126	262/39	13	12.2	19.1	12	-17.7	3.5	Th
CM127-1	262/39	13.3	5.2	17.3	5.5	-20.7	2.5	Th
CM127-2	262/39	13.3	12.1	26.1	10.3	-10.9	3.3	Th
CM128-1	262/39	13.6	7.6	12.9	8.8	-24.7	3.1	Th
CM128-2	262/39	13.6	1.3	19.5	1.3	-19.1	2.6	Th
CM129-1	262/39	13.8	28.2	6.2	32.3	-24.9	7.9	Th
CM129-2	262/39	13.8	9.6	28.2	7.6	-9.3	2.1	Th
CM130-1	262/39	14.1	9	14.9	9.8	-22.5	1.8	Th
CM131-1	262/39	14.3	352.5	7.9	352.5	-31.2	3	Th
CM132-1	262/39	14.5	5.8	8.8	7.7	-29	3.5	Th
CM132-2	262/39	14.5	5.6	18.1	5.7	-19.8	3	Th

CM133-1	262/39	14.6	11.7	6.8	14.7	-29.8	4.9	Th
CM133-2	262/39	14.6	9.5	9.9	11.4	-27.2	2.3	Th
CM134-1	262/39	14.9	9.6	11.1	11.2	-26	6.2	Th
CM134-2	262/39	14.9	7.3	16.4	7.7	-21.3	2	Th
CM135-1	262/39	15	14.6	0.9	19.8	-34.6	4.2	Th
CM136-1	262/39	15.1	9.3	1.5	13.4	-35.5	2.8	Th
CM136-2	262/39	15.1	9.1	6.1	11.9	-31	1.4	Th
CM137-1	262/39	15.4	0.1	10.2	1	-28.4	2.7	Th
CM152	262/41	21.7	184.5	-18.9	184.7	21.2	8.3	Th
CM154	262/41	24	188.1	-4.8	191.6	34.4	13.5	Th
CM155-1	262/41	24.1	194.5	-17.6	194.9	20.4	8.1	Th
CM155-2	262/41	24.1	197.8	-12.6	199.7	24.3	7.4	Th
CM158-1	262/41	24.7	195.5	-7.5	199.2	29.8	4.3	Th
CM158-2	262/41	24.7	208.2	-17.2	208.1	16.4	4	Th
CM159-1	262/41	24.8	190.8	-14.3	192.1	24.5	9.8	Th
CM159-2	262/41	24.8	197.6	-21.7	196.6	15.7	3.1	Th
CM160	262/41	24.9	182.8	-21.1	182.7	19.2	1	Th
CM161-2	262/41	25	196.7	-19.4	196.5	18.1	9.1	Th
CM162-1	262/41	25.2	180.8	-12	181.8	28.6	4.5	Th
CM162-2	262/41	25.2	189.1	-22.7	188.5	16.7	1.9	Th
CM163	262/41	27.2	6.3	34.6	3.8	-5.4	3.1	Th
CM169-2	262/41	28.1	197.7	-21.8	196.7	15.6	4.4	Th

Abbreviations are: Strike/dip, right hand strike and dip of beds; D_g and I_g (D_s and I_s) are declination and inclination in-situ (tilt-corrected), respectively; MAD, Maximum angular deviation from principal component analysis; Demag, demagnetization methods used; Th, Thermal demagnetization.

Supplementary Table 2. Anisotropy of isothermal remanent magnetization of the CORBs samples collected from Cailangba B section.

Site	Sample ID	IRMz/IRMx (acquired between 200 and 1000mT)	IRMz/IRMx Acquired above 650°C	Average IRMz/IRMx Acquired above 650°C
CMC109	CMC109-1	0.6619	0.6176	0.6352
	CMC109-2	0.6644	0.6544	
	CMC109-3	0.6569	0.6224	
	CMC109-4	0.6847	0.6544	
	CMC109-5	0.6519	0.6273	
CMC117	CMC117-1	0.6519	0.6322	0.6343
	CMC117-2	0.6395	0.6273	
	CMC117-3	0.6899	0.6694	
	CMC117-4	0.6694	0.6249	
	CMC117-5	0.6950	0.6176	
CMC124	CMC124-1	0.6273	0.6200	0.6371
	CMC124-2	0.6720	0.6445	
	CMC124-3	0.6847	0.6469	
CMC130	CMC130-1	0.6644	0.6469	0.6820
	CMC130-2	0.7159	0.6873	
	CMC130-3	0.7002	0.6594	
	CMC130-4	0.7454	0.7186	
	CMC130-5	0.6976	0.6976	
CMC137	CMC137-1	0.7054	0.6420	0.6626
	CMC137-2	0.7373	0.7346	
	CMC137-3	0.6771	0.6694	
	CMC137-4	0.7028	0.6371	
	CMC137-5	0.7028	0.6297	

Sample-mean inclination: 25.9°
Average IRMz/IRMx (acquired above 650°C) of sampling section: 0.6502
Corrected site-mean inclination: 36.6°

Abbreviations are: IRMz, Isothermal remanent magnetization of perpendicular bedding. IRMx, Isothermal remanent magnetization of parallel bedding.

Supplementary Table 3. Anisotropy of isothermal remanent magnetization of the Sangdanlin red siliceous shales.

Site	Sample ID	I_{obs} (°)	IRMz/IRMx (acquired between 200 and 800 mT)	IRMz/IRMx Acquired above 650 ℃	Average IRMz/IRMx Acquired above 650 ℃
SES8	SES8-1	23.7	0.7481	0.8127	0.8256
	SES8-2		0.8511	0.8098	
	SES8-3		1.0000	0.9827	
	SES8-4		0.7646	0.7319	
	SES8-5		0.7373	0.8098	
	SES8-6		0.9930	0.9759	
	SES8-7		0.7400	0.6976	
	SES8-8		0.8693	0.7841	
SES9	SES9-1	21.2	0.7373	0.7054	0.6682
	SES9-2		0.7618	0.6569	
	SES9-3		0.6694	0.6104	
	SES9-4		0.7028	0.6796	
	SES9-5		0.9523	0.8511	
	SES9-6		0.7373	0.5774	
	SES9-7		0.7054	0.6445	
	SES9-8		0.7002	0.6200	
SES10	SES10-1	29	0.6899	0.5317	0.6322
	SES10-2		0.8302	0.6544	
	SES10-3		0.8243	0.6224	
	SES10-4		0.8451	0.7841	
	SES10-5		0.7785	0.5727	
	SES10-6		0.8693	0.6494	
	SES10-7		0.7212	0.6249	
	SES10-8		0.8040	0.6176	
SES13	SES13-1	12.8	0.7427	0.6080	0.6671
	SES13-2		0.7536	0.6720	
	SES13-3		0.7133	0.7159	
	SES13-4		0.7133	0.6494	
	SES13-5		0.7028	0.6494	
	SES13-6		0.7926	0.7159	
	SES13-7		0.6297	0.6346	
	SES13-8		0.6924	0.6371	
	SES13-9		0.7028	0.7212	

Site-mean inclination: 18.8 º

Average IRMz/IRMx (acquired above 650 ℃) of sampling section: 0.6982

Corrected site-mean inclination: 26.0 º

Abbreviations are: I_{obs}, Observed mean inclination of sampling sites. IRMz, Isothermal remanent magnetization of perpendicular bedding. IRMx, Isothermal remanent magnetization of parallel bedding.

Supplementary Table 4. The selected Cretaceous and Paleogene paleomagnetic poles from the Lhasa and Tethyan Himalaya terranes.

ID	Site (°N/°E)	Time (Ma)	N/n	Observed Dec (°)	Observed Inc (°)	α_{95} (°)	Paleopole Plat (°)	Paleopole Plon (°)	Paleopole A_{95} (dp/dm)	Paleolat (°)	Rock type	Reliability criteria 1	2	3	4	5	6	7	Q	Inclination shallowing correction	Rock magnetic analysis	Reference
**Lhasa terrane**					**At Reference Position (29.3 °N, 85.3 °E)**																	
1	30.0/91.1	E(53)	9/-	15.4	27.2	9.7	68.9	225.4	7.8	12.5±7.8	Dy	+	+	+	+(F)	+	+	+	7	-	+	Liebke et al. [27]
2	30.0/91.1	E(54-47)	24/195	12.5	39.4	5.3	76.4	212.6	5	20.6±5.0	V	+	+	+	+(F)	+	+	+	7	-	+	Dupont-Nivet et al. [28]
3	30.0/91.0	E	8/46	350.9	25.5	11	71.4	299.8	11	13.6±11	V	+	+	+	+(F)	+	+	+	7	-	-	Achache et al. [29]
4	30.1/90.9	P-E(55)	14/99	359	26.1	9.2	73.6	274.3	7.3	13.1±7.3	V	+	+	+	+(F,R)	+	+	+	7	-	+	Sun et al. [30]
5	29.9/84.3	P-E(57-54)	-/62	348.1	42	7.1	78	329	5.9	23.5±5.9	S	+	+	+	+(F)	+	-	+	6	CV	+	Meng et al. [31]
6	30.0/91.1	E(50-44)	18/-	12.3	23.2	7.2	69.1	234.2	5.6	11.0±5.6	V	+	+	+	+(F)	+	+	+	7	-	+	Chen et al. [32]
7	31.3/95.9	K2	15/-	0.9	24.3	5.6	71.4	273.1	5.2	10.9±5.2	Rb	+	+	+	+(F)	+	-	+	6	A	+	Tong et al. [33]
8	31.2/84.7	K2	33/291	316.8	30.2	5.4	49	344.3	5.3	15.1±5.3	Rb	+	+	+	+(F)	+	-	+	6	CV	+	Yang et al. [34]
9	29.9/90.7	K2	20/126	350.8	32.1	8.1	75	306.7	6.8	17.7±6.8	Rb+V	+	+	+	+(F)	+	-	+	6	CV	+	Sun et al. [35]
10	29.9/91.2	K2	43/377	350.2	23.5	2.5	70.2	300.5	2.2	12.7±2.2	Rb	+	+	+	+(F)	+	+	+	7	-	-	Tan et al. [36]
11	31.6/82.2	K2(~80)	15/-	346.6	25.6	3.5	68.4	298.8	2.7	10.8±2.7	V	+	+	+	+(F)	+	-	+	6	-	+	Yi et al. [37]
12	32.4/80.1	K2(~92)	10/-	21.1	26.8	10	64.1	209	9.6	13.2±9.6	V	+	+	+	+(F,R)	+	+	+	7	-	+	Yi et al. [37]
13	29.9/91.1	K2(~70)	21/-	0.5	20.2	6.4	70.5	269.6	4.9	9.8±4.9	Rb+V	+	+	+	+(F,R)	+	+	+	7	CV	+	Cao et al. [38]
14	32.3/80.1	K2(67-72)	17/-	43.3	30.3	6.9	47.8	181.4	6.4	17.5±6.4	V	+	+	+	+(F)	+	-	+	6	-	+	Ma et al. [39]
15	31.1/84.4	K1(121-117)	12/116	350.5	25.5	7.7	70.5	292.9	7.4	11.7±7.4	V	+	+	+	+(F)	+	-	+	6	-	+	Yang et al. [34]
16	32.3/82.6	K1(132-120)	51/444	28.2	34.5	2.3	61.4	192.9	2.1	17.7±2.1	V	+	+	+	+(F)	+	+	+	7	-	+	Ma et al. [40]
17	31.3/85.1	K1(130-110)	18/162	327	35.7	4.5	58.2	341.9	4.6	18.0±4.6	V	+	+	+	+(F,R)	+	+	+	7	-	+	Chen et al. [41]
18	31.3/91.9	K1(~120.2)	19/163	356.4	16.4	6.3	66.9	281.2	6.1	7.0±6.1	S+V	+	+	+	+(F)	+	+	+	7	-	+	Li et al. [42]
19	32.2/80.4	K1(116-113)	19/164	340.9	36	4.6	69.1	319.8	4.8	16.1±4.8	V+Li	+	+	+	+(F)	+	-	+	6	CV	+	Bian et al. [43]
**Tethyan Himalaya terrane**					**At Reference Position (29.3 °N, 85.3 °E)**																	
1	28.3/88.5	P(59–56)	14/142	357	19.6	3.5	71.6	277.8	2.5	11.3±2.5	Li	+	+	+	+(F,R)	+	+	+	7	-	+	Yi et al. [44]
2	28.3/88.5	P(62–59)	18/171	0.8	11.1	4.2	67.3	266.3	3.5	6.6±3.5	Li	+	+	+	+(F,R)	+	+	+	7	-	+	Yi et al. [44]
3	28.3/88.5	P(63–55)	14/113	356.2	7.9	7.5	65.4	277.6	3.8/7.6	5.2±3.8	Li	+	+	+	+(F,R)	+	+	+	7	-	+	Patzelt et al. [45]

4	28.7/87.2	P	4/28	335.5	–5.9	10.6	50.6	307.9	5.3/10.6	-1.7±5.3	Li	+	-	+	+(F)	+	-	+	5	-	-	Besse et al. [46]
5	28.7/86.8	P	3/15	162.4	29.1	6.6	42.6	280.1	4.0/7.3	-16.8±4.0	Li	+	-	+	+(F)	+	-	+	5	-	-	Tong et al. [47]
**6**	**29.3/85.3**	**P(62.5-59.2)**	**-/86**	**176.3**	**-26**	**2.6**	**74**	**278.5**	**2.5**	**13.7±2.5**	**Rb**	**+**	**+**	**+**	**+(F,R)**	**+**	**+**	**+**	**7**	**A**	**+**	**This study**
7	28.3/88.5	K2(71–65)	14/156	4	-11.2	8.5	55.8	261.4	4.4/8.6	-4.8±4.4	Li	+	+	+	+(F,R)	+	+	+	7	-	+	Patzelt et al. [45]
**8**	**28.9/89.2**	**K2(76.2–74.0)**	**-/127**	**10.3**	**-35**	**2.1**	**40.8**	**256.3**	**1.8**	**-19.4±1.8**	**Rb**	**+**	**+**	**+**	**+(R)**	**+**	**+**	**+**	**7**	**E/I,A**	**+**	**This study**
9	29.7/84.0	K1(120.8–93.9)	12/53	341.7	-51.7	4.7	25	285.7	4.8/7.1	-32.3±4.8	Rb	+	+	+	+(R)	+	+	+	7	A	+	Qin et al. [48]
10	28.1/92.4	K1(134–131)	31/225	261.6	-68.5	3.6	-26.8	315.2	5.7	-46.2±5.7	V	+	+	+	+(F,R)	+	+	+	7	-	+	Yang et al. [49]
11	28.8/91.3	K1(135–124)	26/216	296.1	-65.7	4	-5.9	308	6.1	-43.5±6.1	V	+	+	+	+(F,R)	+	+	+	7	-	+	Ma et al. [50]
12	28.9/91.3	K1(138–135)	31/-	321.2	-68.7	4.4	0.9	293.4	7	-49.6±7.0	V	+	+	+	+(F,R)	+	+	+	7	-	+	Bian et al. [51]
13	28.8/83.8	K1	-/95	326	-61.5	5	12	288.7	6.0/7.5	-42.9±6.0	S	+	+	+	-	+	-	+	5	-	-	Klootwijk and Bingham [52]
14	28.5/87.0	K1	-/201	19.7	-71	3	4.4	256	3	-55.1±3.0	S	+	+	+	-	+	-	+	5	-	+	Huang et al. [53]
15	28.1/92.4	K1(~131.1)	8/-	7.3	-56.8	16.1	22	266.7	7.6	-38.7±7.6	V	+	+	+	+(F)	+	+	+	7	-	+	Meng et al. [54]
16	28.1/92.4	K1	20/-	6.4	-57.8	6					Li	+	+	+	+(F,R)	+	+	+	7	E/I	+	Meng et al. [54]
17	29.7/83.6	K1	-/53	3.1	-47.8	4.4	29.7	260.1	4.9	-30.8±4.9	S+Ch	+	+	+	+(F,R)	+	+	+	7	E/I	+	Meng et al. [54]
**India APWP**				**At Reference Position (29.3°N, 85.3°E)**																		
1		40					74.7	286.8	2.9	14.9±2.9												Torsvik et al. [55]
2		50					65.1	278.4	2.8	4.9±2.8												Torsvik et al. [55]
3		60					48.5	280.9	2.1	-11±2.1												Torsvik et al. [55]
4		70					36.4	280.7	2.5	-22.7±2.5												Torsvik et al. [55]
5		80					29.1	283.5	2.9	-29.1±2.9												Torsvik et al. [55]
6		90					20.9	291.4	2.5	-33.9±2.5												Torsvik et al. [55]
7		100					19.7	293	3.3	-34.2±3.3												Torsvik et al. [55]
8		110					11	295.9	3.3	-40±3.3												Torsvik et al. [55]
9		120					8.6	296.4	2.6	-41.7±2.6												Torsvik et al. [55]
10		130					-1.1	297.1	2.8	-48.6±2.8												Torsvik et al. [55]

Abbreviations are: ID, Serial number; Time (Ma): K2, Late Cretaceous. K1, Early Cretaceous. P, Paleocene. E, Eocene; n/N, number of samples or sites used to calculate Fisher mean; Dec and Inc are declination and inclination, respectively; Plat and Plong are latitude and longitude of

paleopoles; α_{95} and A_{95} is the radius of cone at 95% confidence level about the mean direction, A_{95} = (dp*dm)^0.5; Paleolat is the paleolatitude calculated for the reference point at 29.3 °N, 85.3 °E; Rock type: Rb, Red-beds; S, sandstone; Li, limestone; V, volcanic rock; D, dyke; Ch, chert. Data reliability criteria is after: 1, well-determined rock age and a presumption that magnetization is the same age; 2, sufficient quantity of samples (n > 50 specimens or N > 5 sites) and adequate statistical precision, k (or K) ≥ 10 and α_{95} (A_{95}) ≤ 16.0; 3, detailed demagnetization and isolation of magnetic components; 4, positive field stability tests; 5, structural control and tectonic coherence with craton or block involved; 6, presence of reversals; 7, no resemblance to paleopoles of younger age (by more than a period) [56]. Q, the reliability factor; "+" in the criterion column means fulfilling this criterion; "-" in the criterion column means failing to fulfill this criterion. Field tests: F means positive fold test, R means positive reversal test; Inclination shallowing correction: E/I, E/I inclination shallowing correction; A, anisotropy-based inclination correction; CV, consistent with the paleomagnetic data obtained from volcanic rocks in the same study; Rock magnetic analysis,"+" means fulfilling this criterion; "-"means failing to fulfill this criterion.

Supplementary References

1. Yin A and Harrison TM. Geologic evolution of the Himalayan-Tibetan orogen. *Annu Rev Earth Planet Sci* 2000; **28**: 211–280.
2. Ding L, Kapp P and Wan X. Paleocene–Eocene record of ophiolite obduction and initial India-Asia collision, south central Tibet. *Tectonics* 2005; **24**: TC3001.
3. Burg JP, Leyreloup A and Girardeau J *et al.* Structure and metamorphism of a tectonically thickened continental crust: The Yalu Tsangpo suture (Tibet). *Philos Trans R Soc Lond* 1987; **321**: 67–86.
4. An W, Hu X and Garzanti E *et al.* Xigaze forearc basin revisited (South Tibet): Provenance changes and origin of the Xigaze Ophiolite. *Geol Soc Am Bull* 2014; **126**: 1595–1613.
5. Liu G and Einsele G. Sedimentary history of the Tethyan basin in the Tibetan Himalayas. *Geol Rundsch* 1994; **83**: 32–61.
6. Chen X, Wang C and Kuhnt W *et al.* Lithofacies, microfacies and depositional environments of Upper Cretaceous Oceanic red beds (Chuangde Formation) in southern Tibet. *Sed Geol* 2011; **235**: 100–110.
7. Li X, Wang C and Hu X. Stratigraphy of deep-water Cretaceous deposits in Gyangze, southern Tibet, China. *Cretaceous Res* 2005; **26**: 33–41.
8. Wan X, Lamolda MA and Si J *et al.* Foraminiferal stratigraphy of Late Cretaceous red beds in southern Tibet. *Cretaceous Res* 2005; **26**: 43–48.
9. Hu X, Wang C and Li X *et al.* Upper Cretaceous oceanic red beds in southern Tibet: Lithofacies, environments and colour origin. *Sci China Ser D-Earth Sci* 2006; **49**: 785–795.
10. DeCelles PG, Kapp P and Gehrels GE *et al.* Paleocene–Eocene foreland basin evolution in the Himalaya of southern Tibet and Nepal: implications for the age of

initial India-Asia collision. *Tectonics* 2014; **33**: 824–849.

11. Ding L, Kapp P and Wan X. Paleocene–Eocene record of ophiolite obduction and initial India-Asia collision, south central Tibet. *Tectonics* 2005; **24**: TC3001.
12. Wang J, Hu X and Jansa L *et al.* Provenance of the Upper Cretaceous-Eocene deep-water sandstones in Sangdanlin, southern Tibet: Constraints on the timing of initial India-Asia collision. *J Geol* 2011; **119**: 293–309.
13. Hu X, Garzanti E and Moore T *et al.* Direct stratigraphic dating of India-Asia collision onset at the Selandian (middle Paleocene, 59 ± 1 Ma). *Geology* 2015; **43**: 859–862.
14. Wu FY, Ji WQ and Wang JG *et al.* Zircon U-Pb and Hf isotopic constraints on the onset time of India-Asia collision. *Am J Sci* 2014; **314**: 548–579.
15. Li S, Deng C and Paterson GA *et al.* Tectonic and sedimentary evolution of the late Miocene–Pleistocene Dali Basin in the southeast margin of the Tibetan Plateau: Evidences from anisotropy of magnetic susceptibility and rock magnetic data. *Tectonophysics* 2014; **629**: 362–377.
16. McElhinny MW. Statistical significance of the fold test in paleomagnetism. *Geophys J R Astron Soc* 1964; **8**: 338–340.
17. McFadden PL. A new fold test for paleomagnetic studies. *Geophys J Int* 1990; **103**: 163–169.
18. Watson GS and Enkin RJ. The fold test in paleomagnetism as a parameter estimation problem. *Geophys Res Lett* 1993; **20**: 2135–2137.
19. Tong Y, Yang Z and Mao C *et al.* Paleomagnetism of Eocene red-beds in the eastern part of the Qiangtang Terrane and its implications for uplift and southward crustal extrusion in the southeastern edge of the Tibetan Plateau. *Earth Planet Sci Lett* 2017; **475**: 1–14.

20. Ingalls M, Rowley DB and Currie B *et al.* Large-scale subduction of continental crust implied by India–Asia mass-balance calculation. *Nat Geosci* 2016; **9**: 848–853.
21. Meng J, Gilder SA and Wang C *et al.* Defining the limits of Greater India. *Geophys Res Lett* 2019; **46**: 4182–4191.
22. van Hinsbergen DJJ, Lippert PC and Dupont-Nivet G *et al.* Greater India Basin hypothesis and a two-stage Cenozoic collision between India and Asia. *Proc Natl Acad Sci USA* 2012; **109**: 7659–7664.
23. van Hinsbergen DJJ, Lippert PC and Li S *et al.* Reconstructing Greater India: Paleogeographic, kinematic, and geodynamic perspectives. *Tectonophysics* 2019; **760**: 69–94.
24. Aitchison JC, Ali JR and Davis AM. When and where did India and Asia collide? *J Geophys Res* 2007; **112**: B05423.
25. Kapp P and DeCelles PG. Mesozoic–Cenozoic geological evolution of the Himalayan-Tibetan orogen and working tectonic hypotheses. *Am J Sci* 2019; **319**: 159–254.
26. Gradstein FM, Ogg JG and Schmitz M *et al.* *The Geologic Time Scale 2012.* Amsterdam: Elsevier, 2012.
27. Liebke U, Appel E and Ding L *et al.* Position of the Lhasa terrane prior to India-Asia collision derived from palaeomagnetic inclinations of 53 Ma old dykes of the Linzhou Basin: constraints on the age of collision and postcollisional shortening within the Tibetan Plateau. *Geophys J Int* 2010; **182**: 1199–1215.
28. Dupont-Nivet G, Lippert PC and van Hinsbergen DJJ *et al.* Paleolatitude and age of the Indo-Asia collision: paleomagnetic constraints. *Geophys J Int* 2010; **182**: 1189–1198.

29. Achache J, Courtillot V and Zhou YX. Paleogeographic and tectonic evolution of southern Tibet since middle Cretaceous time: New paleomagnetic data and synthesis. *J Geophys Res* 1984; **89**: 10,311–10,339.

30. Sun Z, Jiang W and Li H *et al.* New paleomagnetic results of Paleocene volcanic rocks from the Lhasa block: tectonic implications for the collision of India and Asia. *Tectonophysics* 2010; **490**: 257–266.

31. Meng J, Wang C and Zhao X *et al.* India-Asia collision was at 24°N and 50 Ma: palaeomagnetic proof from southernmost Asia. *Sci Rep* 2012; **2**: 925.

32. Chen J, Huang B and Yi Z *et al.* Paleomagnetic and ^{40}Ar/^{39}Ar geochronological results from the Linzizong group, Linzhou Basin, Lhasa Terrane, Tibet: implications to Paleogene paleolatitude and onset of the India–Asia collision. *J Asian Earth Sci* 2014; **96**: 162–177.

33. Tong Y, Yang Z and Pei J *et al.* Paleomagnetism of the Upper Cretaceous red-beds from the eastern edge of the Lhasa Terrane: New constraints on the onset of the India-Eurasia collision and latitudinal crustal shortening in southern Eurasia. *Gondwana Res* 2017; **48**: 86–100.

34. Yang T, Ma Y and Zhang S *et al.* New insights into the India-Asia collision process from Cretaceous paleomagnetic and geochronologic results in the Lhasa terrane. *Gondwana Res* 2015; **28**: 625–641.

35. Sun Z, Pei J and Li H *et al.* Palaeomagnetism of late Cretaceous sediments from southern Tibet: evidence for the consistent palaeolatitudes of the southern margin of Eurasia prior to the collision with India. *Gondwana Res* 2012; **21**: 53–63.

36. Tan X, Gilder S and Kodama KP *et al.* New paleomagnetic results from the Lhasa block: revised estimation of latitudinal shortening across Tibet and implications for dating the India-Asia collision. *Earth Planet Sci Lett* 2010; **293**: 396–404.

37. Yi Z, Huang B and Yang L *et al.* A quasi-linear structure of the southern margin of Eurasia prior to the India-Asia collision: First paleomagnetic constraints from Upper Cretaceous volcanic rocks near the western syntaxis of Tibet. Tectonics 2015; **34**: 1431–1451.

38. Cao Y, Sun Z and Li H *et al.* New Late Cretaceous paleomagnetic data from volcanic rocks and red siliceous shales from the Lhasa terrane and its implications for the paleolatitude of the southern margin of Asia prior to the collision with India. *Gondwana Res* 2017; **41**: 337–351.

39. Ma Y, Yang T and W. Bian *et al.* Paleomagnetic and geochronologic results of latest Cretaceous lava flows from the Lhasa terrane and their tectonic implications. *J Geophys Res Solid Earth* 2017; **122**: 8786–8809.

40. Ma Y, Yang T and Yang Z *et al.* Paleomagnetism and U-Pb zircon geochronology of Lower Cretaceous lava flows from the western Lhasa terrane: new constraints on the India-Asia collision process and intracontinental deformation within Asia. *J Geophys Res Solid Earth* 2014; **119**: 7404–7424.

41. Chen W, Yang T and Zhang S *et al.* Paleomagnetic results from the Early Cretaceous Zenong Group volcanic rocks, Cuoqin, Tibet, and their paleogeographic implications. *Gondwana Res* 2012; **22**: 461–469.

42. Li Z, Ding L and Song P *et al.* Paleomagnetic constraints on the paleolatitude of the Lhasa block during the Early Cretaceous: Implications for the onset of India-Asia collision and latitudinal shortening estimates across Tibet and stable Asia. *Gondwana Res* 2017; **41**: 352–372.

43. Bian W, Yang T and Ma Y *et al.* New Early Cretaceous palaeomagnetic and geochronological results from the far western Lhasa terrane: Contributions to the Lhasa-Qiangtang collision. *Sci Rep* 2017; **7**: 16216.

44. Yi Z, Huang B and Chen J *et al.* Paleomagnetism of early Paleogene marine sediments in southern Tibet, China: Implications to onset of the India-Asia collision and size of Greater India. *Earth Planet Sci Lett* 2011; **309**: 153–165.

45. Patzelt A, Li H and Wang J *et al.* Palaeomagnetism of Cretaceous to Tertiary sediments from southern Tibet: evidence for the extent of the northern margin of India prior to the collision with Eurasia. *Tectonophysics* 1996; **259**: 259–284.

46. Besse J, Courtillot V and Pozzi JP *et al.* Paleomagnetic estimates of crustal shortening in the Himalayan thrusts and Zangbo suture. *Nature* 1984; **311**: 621–626.

47. Tong Y, Yang Z and Zheng L *et al.* Early Paleocene paleomagnetic results from southern Tibet, and tectonic implications. *Int Geol Rev* 2008; **50**: 546–562.

48. Qin SX, Li YX and Li XH *et al.* Paleomagnetic results of Cretaceous cherts from Zhongba, southern Tibet: New constraints on the India-Asia collision. *J Asian Earth Sci* 2019; **173**: 42–53.

49. Yang T, Ma Y and Bian W *et al.* Paleomagnetic results from the Early Cretaceous Lakang Formation lavas: constraints on the paleolatitude of the Tethyan Himalaya and the India–Asia collision. *Earth Planet Sci Lett* 2015; **428**: 120–133.

50. Ma Y, Yang T and Bian W *et al.* Early Cretaceous paleomagnetic and geochronologic results from the Tethyan Himalaya: Insights into the Neotethyan paleogeography and the India–Asia collision. *Sci Rep* 2016; **6**: 21605.

51. Bian W, Yang T and Ma Y *et al.* Paleomagnetic and geochronological results from the Zhela and Weimei Formations lava flows of the eastern Tethyan Himalaya: New insights into the breakup of eastern Gondwana. *J Geophys Res Solid Earth* 2019; **124**: 44–64.

52. Klootwijk CT and Bingham DK. The extent of greater India, III. Palaeomagnetic

data from the Tibetan Sedimentary series, Thakkhola region, Nepal Himalaya. *Earth Planet Sci Lett* 1980; **51**: 381–405.

53. Huang W, van Hinsbergen DJJ and Dekkers MJ *et al.* Paleolatitudes of the Tibetan Himalaya from primary and secondary magnetizations of Jurassic to Lower Cretaceous sedimentary rocks. *Geochem Geophys Geosyst* 2015; **16**: 77–100.
54. Meng J, Gilder SA and Wang C *et al.* Defining the Limits of Greater India. *Geophys Res Lett* 2019; **46**: 4182–4191.
55. Torsvik TH, Müller RD and van der Voo R *et al.* Phanerozoic polar wander, palaeogeography and dynamics. *Earth-Sci Rev* 2012; **114**: 325–368.
56. van der Voo R. The reliability of paleomagnetic data. *Tectonophysics* 1990; **184**: 1–9.
